# An Energy-Equivalent *d^+^*/*d^−^* Damage Model with Enhanced Microcrack Closure-Reopening Capabilities for Cohesive-Frictional Materials

**DOI:** 10.3390/ma10040433

**Published:** 2017-04-20

**Authors:** Miguel Cervera, Claudia Tesei

**Affiliations:** 1 International Center for Numerical Methods in Engineering (CIMNE), Universidad Politécnica de Cataluña, Campus Norte UPC, 08034 Barcelona, Spain; miguel.cervera@upc.edu; 2 Department of Structural, Building and Geotechnical Engineering (DISEG), Politecnico di Torino, Corso Duca degli Abruzzi 24, 10129 Torino, Italy

**Keywords:** cohesive-frictional materials, damage-induced orthotropy, microcrack closure-reopening effects, cyclic loading, energy equivalence, spectral decomposition

## Abstract

In this paper, an energy-equivalent orthotropic *d^+^*/*d^−^* damage model for cohesive-frictional materials is formulated. Two essential mechanical features are addressed, the damage-induced anisotropy and the microcrack closure-reopening (MCR) effects, in order to provide an enhancement of the original *d^+^*/*d^−^* model proposed by Faria et al. 1998, while keeping its high algorithmic efficiency unaltered. First, in order to ensure the symmetry and positive definiteness of the secant operator, the new formulation is developed in an energy-equivalence framework. This proves thermodynamic consistency and allows one to describe a fundamental feature of the orthotropic damage models, i.e., the reduction of the Poisson’s ratio throughout the damage process. Secondly, a “multidirectional” damage procedure is presented to extend the MCR capabilities of the original model. The fundamental aspects of this approach, devised for generic cyclic conditions, lie in maintaining only two scalar damage variables in the constitutive law, while preserving memory of the degradation directionality. The enhanced unilateral capabilities are explored with reference to the problem of a panel subjected to in-plane cyclic shear, with or without vertical pre-compression; depending on the ratio between shear and pre-compression, an absent, a partial or a complete stiffness recovery is simulated with the new multidirectional procedure.

## 1. Introduction

The design of constitutive models adequate for predicting the structural response of cohesive-frictional materials continues to be a challenging topic in the civil engineering field because cementitious materials, such as concrete, rocks and masonry, can be classified within this category. 

The non-linear behavior of cohesive-frictional materials is mainly related to damage and plasticity phenomena. Relatively simple damage formulations, as the one proposed in this paper, are able to provide a reliable mechanical response for a great variety of loading conditions (as observed in [1]) requiring a small number of constitutive parameters. Some relevant contributions in the field of continuum damage mechanics applied to cohesive-frictional materials are, among others, the ones presented in [1,2,3,4,5,6]. Furthermore, several formulations have been developed within the framework of damage combined with plasticity (see [7,8,9,10,11,12,13,14]).

Continuum damage mechanics is based on the introduction of suitable internal variables in the constitutive law with the aim of simulating the elastic stiffness degradation and the strength decrease associated with the growth of microvoids and microcracks in the material, taking into account the irreversibility of the thermodynamic processes. Common key ingredients of the several damage formulations are the concepts of effective (undamaged) configuration and nominal (damaged) one, as well as the hypotheses of strain equivalence [8,15], stress equivalence [8] or energy equivalence [16,17], by means of which the relations between effective and nominal configurations are established. 

Despite the abundant research work delving into the topic of continuum damage mechanics, some specific aspects related to its application to cohesive-frictional materials require further investigation. The objective of the present paper is an enhancement of the *d^+^*/*d^−^* formulation first introduced in [5,18,19], deeply developed in [1] and then extended to plasticity in [12], hereafter termed “the original *d^+^*/*d^−^* model”. The interest for this model derives from the fact that it combines an adequate non-linear structural response and algorithmic efficiency, its implementation and use in standard FE codes being relatively easy. Confirmation of its effectiveness can be found in the several structural applications in which it has been satisfactorily adopted: for the seismic analysis of concrete dams [1], in the assessment of reinforced concrete walls subjected to seismic shear loadings [20], for the characterization of the in-plane [21] and out-of-plane [22] behavior of masonry panels, in the evaluation of historical masonry structures [23] and in the macro-modelling of masonry, combined with a tensor mapping procedure [24,25].

The essential mechanical features for the description of cohesive-frictional materials are the following:

1. degradation of the elastic stiffness and softening response in the post-peak regime, with reduction of the peak strength for increasing deformation levels;
2. non-symmetrical material behavior between tension and compression due to different strengths and different fracture energies;
3. anisotropy induced in the material by the damage process, due to nucleation and evolution of planar microvoids “in the planar direction perpendicular to the maximum tensile strain” [26]. Hence, except for the case of hydrostatic stress or strain conditions, isotropic models are incomplete in the description of damage, which is intrinsically an anisotropic phenomenon [27] and drastically neglects the possibility of strut action in the assessment of the structural capacity [28];
4. microcrack closure-reopening (MCR) effects, i.e., partial or total stiffness recovery in the transition from tension to compression, crucial in cyclic conditions, experimentally documented for concrete in [29].

Both Properties (1) and (2) are successfully satisfied by the original *d^+^*/*d^−^* damage model; specifically, the asymmetrical performance of the material under tensile and compressive regimes is modelled by means of only two scalar variables, *d^+^* and *d^−^*, in combination with the spectral decomposition of a suitable second order tensor (the effective stress one). The high algorithmic efficiency of the model lies in this adoption of two scalar quantities for the representation of damage and in the recourse to a strain-driven formalism (strictly related to the choice of splitting the effective stress in a strain-equivalence framework).

Although Features (3) and (4) are taken into account by the original *d^+^*/*d^−^* damage model, in the present work, these capabilities are enhanced by proposing a new *d^+^*/*d^−^* formulation, which represents the damage-induced anisotropy in an energy-equivalent framework and not in a strain-equivalent one. Moreover, a “multidirectional” damage approach is developed to extend the microcrack closure-reopening capabilities of the original model to generic cyclic conditions, particularly improving the material response under shear cyclic loading.

Regarding Point (3), on the one hand, the basic idea of the original model to simulate the directional degradation through the use of a spectral decomposition is maintained, with the consequent acceptance of a perfect coincidence between the axes of anisotropy (in particular orthotropy) of the damaged material and the strain and stress principal directions. On the other hand, an explicit secant stiffness matrix representing the damage-induce anisotropy is here provided. The explicit definition of the secant matrix is useful for the implementation of Picard’s method, which is more robust than the Newton–Raphson one, even though it does not show quadratic convergence. Thermodynamic and practical reasons require the secant stiffness operator to be positive definite and endowed with both major and minor symmetries. The fulfilment of these features leads to the formulation of a new *d^+^*/*d^−^* damage model based on the energy equivalence assumption. Such a choice ensures full thermodynamic consistency and reflects in the formulation of a stable orthotropic damaged material. Moreover, it translates in an adequate consideration of the Poisson’s effect and, specifically, in the simulation of a nominal Poisson’s ratio, which does not remain constant throughout the damage process.

Regarding Point (4), a shortcoming of the original model is identified in its incapability of maintaining permanent memory of the damage orientation. Although the original model succeeds in modelling the regain of stiffness from tension to compression in a uniaxial cyclic loading history, in generic cyclic loading conditions, as shear, involving the coexistence of compressive and tensile regimes, the just underlined limitation affects the structural response unrealistically. In this regard, in the present work, the microcrack closure-reopening capabilities of the original model are extended to generic cyclic conditions by the formulation of a “multidirectional” damage model. This concept consists of saving during the analyses two damage values for tension and two damage values for compression, differing for the principal strain directions that have generated them and in choosing the active *d^+^* (*d^−^*) depending on the current maximum (minimum) principal strain direction. Inactive values are however maintained with the possibility of being-reactivated in the occurrence of a principal directions’ rotation. The “multidirectional” damage procedure allows for the preservation of memory about damage orientation while keeping unaltered the scalar damage nature of the original model.

The present paper is structured as follows. In Section 2, the salient aspects of the original *d^+^*/*d^−^* model are briefly recalled, and different possible definitions for the secant stiffness matrix in a strain equivalence framework are explored. Then, in Section 3, the new energy-equivalent damage formulation is presented and its thermodynamic consistency discussed. Moreover, the damage criteria and the damage evolution laws adopted for the definition of *d^+^* and *d^−^* are provided. Appendix A completes this section with the proof of the second principle of thermo-dynamics for the new model. In Section 4, the multidirectional procedure is extensively described with reference to plane problems. Reflections on how to extend the formulation to the 3D case are however added. To demonstrate the enhancements in terms of both Poisson’s effect and stiffness recovery capabilities under cyclic conditions, some examples solved with the original and the new *d^+^*/*d^−^* damage formulation are shown in Section 5. These betterments are finally summarized in Section 6, which is dedicated to the concluding remarks.

### Notation

***I*_2_** and $I=(I_{2}\bar{\underline{⊗}}I_{2})$ are the second and fourth order identity symmetric tensors, respectively.

## 2. Original *d^+^*/*d^−^* Damage Model

### 2.1. Strain Equivalence and Constitutive Law

The original *d^+^*/*d^−^* damage model is based on the notion of effective stress and on the hypothesis of strain equivalence. The effective stress $\bar{\sigma }$ and the effective strain $\bar{\varepsilon }$ are the stress and strain to which the undamaged material between micro-cracks is subjected; they are related by the fourth-order elastic tensor ***D*_0_**:

(1) $$\bar{\sigma }=D_{0}:\bar{\varepsilon }$$

The nominal quantities σ and ε refer to an average of the corresponding effective quantities on the total surface of the material (including also micro-cracks); for instance, the nominal stress tensor is related to the effective one by means of a fourth-order tensor ***A*** dependent on damage:

(2) $$\sigma =A:\bar{\sigma }$$

The strain equivalence assumption, as formulated in [8,15], asserts that: “The strain associated with a damage state under the applied stress ***σ*** is equivalent to the strain associated with its undamaged state under the effective stress $\bar{\sigma }$”. In other words, it is considered by hypothesis that the effective and the nominal strain are equal ($\varepsilon =\bar{\varepsilon }$) and that only the nominal and the effective stress are different. Consequently, the effective stress of Equation (1) can be rewritten as:

(3) $$\bar{\sigma }=D_{0}:\varepsilon$$

The basic features of concrete that the original model reproduces are the following ones: (i) the development of irreversible deformations; (ii) the strongly asymmetrical behavior under tension and compression; (iii) the microcrack closure-reopening (MCR) effects visible in the case of uniaxial cyclic actions, i.e., the stiffness recovery when passing from tension to compression and vice versa; and (iv) the anisotropy induced by the damage process in the material.

Since the modelling of the plastic strains is outside the scope of the present study, they are assumed equal to zero, and the elastic strain tensor ***ε_e_*** adopted in the original model is replaced by the total strain ***ε*** henceforth, so that ***ε*** = ***ε_e_***. In order to represent the differences between the stress-strain envelops under tension and under compression, two independent scalar variables, one for tension *d^+^* and one for compression *d^−^*, are introduced. Moreover, to deal with Points (ii), (iii) and (iv), a spectral decomposition of the effective stress tensor (Equation (3)) into a positive and a negative part is carried out:

(4) $${\bar{\sigma }}^{+}=\sum_{i=1}^{3}\langle {\bar{\sigma }}_{i}\rangle p_{i}⊗p_{i}=Q:\bar{\sigma }\;{\bar{\sigma }}^{-}=\bar{\sigma }-{\bar{\sigma }}^{+}=\left(I-Q\right):\bar{\sigma }$$

where ***p_i_*** is the eigenvector identifying the principal direction *i*-th of the effective stress tensor while the Macaulay brackets act on the *i*-th principal value of the effective stress tensor ${\bar{\sigma }}_{i}$ in such a way that: if ${\bar{\sigma }}_{i}$ is positive (tensile principal stress), $\langle {\bar{\sigma }}_{i}\rangle ={\bar{\sigma }}_{i}$; else (compressive principal stress) $\langle {\bar{\sigma }}_{i}\rangle =0$. The fourth-order projection operator ***Q***, which extracts from the effective stress tensor its positive part, is:

(5) $$Q=\sum_{i=1}^{3}H({\bar{\sigma }}_{i})p_{i}⊗p_{i}⊗p_{i}⊗p_{i}$$

and its explicit expression is given in [30]. *H*(${\bar{\sigma }}_{i}$) is the Heaviside function, such that, if ${\bar{\sigma }}_{i}$is positive, *H*(${\bar{\sigma }}_{i}$) = 1; else, *H*(${\bar{\sigma }}_{i}$) = 0.

The constitutive law of the original model is written in terms of the spectral decomposition of the effective stress tensor (4) and has the following expression:

(6) $$\sigma =D_{s}:\varepsilon =\left(1-d^{+}\right){\bar{\sigma }}^{+}+\left(1-d^{-}\right){\bar{\sigma }}^{-}$$

where ***D_S_*** is the fourth-order secant stiffness operator (subscript “*_S_*” stands for strain equivalence), introduced in order to relate the nominal stress tensor ***σ*** to the nominal strain tensor ***ε***.

**Remark** **2.1.** *The versatility of the model in treating the damage-induced anisotropy is intrinsic in Equation (6). In fact, depending on the sign of the principal effective stresses, two different cases can be* *distinguished:*

- ${\bar{\sigma }}_{i}$ *with concordant sign (*$\bar{\sigma }={\bar{\sigma }}^{+}$ *or* $\bar{\sigma }={\bar{\sigma }}^{-}$*): isotropy is preserved after damage, and an isotropic (tensile or compressive, respectively) damage model is recovered.*
- ${\bar{\sigma }}_{i}$ *with discordant sign: the damaged material is anisotropic, and the directions of maximum and minimum axial stiffnesses are coincident with the principal directions of the effective stress tensor. As observed in [31,32], the coaxiality between the reference system of the anisotropic material and the principal directions of the nominal strain **ε** is a particular condition in anisotropic elasticity, which corresponds to the extremization of the strain energy density. In addition, the maximization or minimization of the strain energy density implies the coaxiality between the nominal strain tensor **ε** and the nominal stress tensor **σ**. These observations lead to assert that the d^+^/d^−^ formulation can be interpreted within the rotating smeared crack concept, due to the co-rotation of the axes of material anisotropy with the principal axes of the strain **ε**, which consequently coincide also with the principal axes of the stress **σ**.*

### 2.2. Discussion on Different Secant Stiffness Operators Based on Strain Equivalence

In [1], the constitutive law (6) is provided, but not the definition of the fourth-order secant stiffness tensor ***D_S_*** relating the nominal stress ***σ*** and the nominal strain ***ε***. In this section, this issue is addressed evaluating two different expressions for the secant stiffness ***D_S_***, both derived in the hypothesis of strain equivalence, i.e., considering the effective stress definition shown in Equation (3). In order to guarantee thermodynamic consistency, two properties have to be fulfilled by the secant operator: major symmetry (in addition to the minor ones), as stated in [1] with reference to the Schwartz theorem about the equality of the mixed partial of the potential (6); positive definiteness, a major requirement in order to have a damaged orthotropic material with stable behavior.

The first expression to be considered for the secant stiffness tensor, ***D_S_*_1_**, is obtained by replacing in the constitutive law (6) the positive and negative parts of the effective stress tensor (4), expressed in terms of the positive projection operator ***Q*** (5):

(7) $$\sigma =\left(1-d^{+}\right){\bar{\sigma }}^{+}+\left(1-d^{-}\right){\bar{\sigma }}^{-}=\left(1-d^{+}\right)Q:D_{0}:\varepsilon +\left(1-d^{-}\right)\left(I-Q\right):D_{0}:\varepsilon$$

From Equation (7), exploiting the distributive rule, the expression of ***D_S_*_1_** is:

(8) $$D_{s1}=\left(1-d^{+}\right)Q:D_{0}+\left(1-d^{-}\right)\left(I-Q\right):D_{0}$$

Considering the constitutive law written in terms of ***D_S_*_1_** (8) and referring to the Relations (2) and (3) for the nominal and effective stress tensors, respectively, the definition of the fourth-order operator ***A*** appearing in Equation (2) is:

(9) $$A=\left(1-d^{+}\right)Q+\left(1-d^{-}\right)\left(I-Q\right)$$

Although both the projection operator ***Q*** (5) and the elastic fourth-order tensor ***D*_0_** have major symmetry, this does not imply that the fourth-order tensor ***D_S_*_1_** (8) resulting from their double contraction is necessarily endowed with major symmetry. Specifically, besides the undamaged situation (***D_S_*_1_** = ***D*****_0_**), the symmetry of the secant operator is guaranteed only when the Poisson’s ratio is null or when an isotropic damage model is recovered, i.e., in the cases of purely tensile regimes or purely compressive regimes (see Remark 2.1).

Since the quadratic form associated with a non-symmetric matrix is equal to the quadratic form associated with its symmetric part only, the second expression to be evaluated for the secant operator, ***D_s_*_2_**, is the symmetric part of the non-symmetric secant operator ***D_s_*_1_** (8):

(10) $$D_{s2}=\frac{1}{2}\left(D_{s1}+D_{s1}^{T}\right)=\frac{1}{2}\left(1-d^{+}\right)\left[Q:D_{0}+D_{0}:Q\right]+\frac{1}{2}\left(1-d^{-}\right)\left[\left(I-Q\right):D_{0}+D_{0}:\left(I-Q\right)\right]$$

However, for this second proposal, the positive definiteness cannot be proven. This is shown by resorting to Equation (11), where the matrix form of ***D_S_*_2_** is given in the principal reference system of orthotropy of the damaged material, in terms of the Lamé constants and the damage variables; a 3D stress state composed of two tensile directions and a compressive one is considered in such a way that two eigenvectors contribute in defining ***Q*** (5).

(11) $$\left[\begin{array}{cccccc} \left(1\;-d^{+})(2G+\lambda \right) & \left[\left(1\;-d^{+}\right)\lambda +\;\left(1\;-d^{-}\right)\lambda \right]/2 & \left(1\;-d^{+}\right)\lambda & 0 & 0 & 0\\ \left[\left(1\;-d^{+}\right)\lambda +\left(1\;-d^{-}\right)\lambda \right]/2 & \left(1\;-d^{-}\right)\left(2G+\lambda \right) & \left[\left(1\;-d^{+}\right)\lambda +\;\left(1\;-d^{-}\right)\lambda \right]/2 & 0 & 0 & 0\\ \left(1\;-d^{+}\right)\lambda & \left[\left(1\;-d^{+}\right)\lambda +\;\left(1\;-d^{-}\right)\lambda \right]/2 & \left(1\;-d^{+}\right)\left(2G+\lambda \right) & 0 & 0 & 0\\ 0 & 0 & 0 & \left(1\;-d^{-}\right)G & 0 & 0\\ 0 & 0 & 0 & 0 & \left(1\;-d^{-}\right)G & 0\\ 0 & 0 & 0 & 0 & 0 & \left(1\;-d^{-}\right)G \end{array}\right]$$

Despite the evident symmetry of the matrix (11), it is easy to demonstrate its lack of positive definiteness by adopting Sylvester’s criterion. In fact, analyzing the first principal minor of order two, equal to:

(12) $$\Delta_{2}=\left(1-d^{+}\right)\cdot \left(1-d^{-}\right){\left(2G+\lambda \right)}^{2}-{\left[\left(1-d^{+}\right)+\left(1-d^{-}\right)\right]}^{2}\lambda^{2}/4$$

and considering *d^+^* = 1 and *d^−^* = 0, it follows that the quantity (12) is negative.

Therefore, the necessity of formulating a new version of the original *d^+^*/*d^−^* model derives mainly from the just-described difficulties found in the definition of a consistent secant stiffness operator in a strain-equivalence framework. Consequently, in accordance with the discussion provided in [17] for a general anisotropic damage model, in the new *d^+^*/*d^−^* formulation presented henceforth, the strain equivalence assumption is abandoned in favor of an energy equivalence one.

## 3. New *d^+^*/*d^−^* Damage Model Based on Energy Equivalence

### 3.1. Consistent Secant Stiffness for the Damage-Induced Orthotropy

The hypothesis of energy equivalence (see [16,17]) consists of considering the coincidence between the energy stored in the terms of the nominal quantities and secant stiffness and the elastic energy stored in terms of the effective quantities and undamaged linear elastic stiffness. This means that neither the nominal stress, nor the nominal strain are equal to their effective counterparts; therefore, in addition to the relation (2) between the nominal and the effective stress, a relation between the effective and the nominal strain is needed. Following the procedure described in [17], this relation is governed by the fourth-order tensor ***A*** introduced in Equation (2) and is written as:

(13) $$\bar{\varepsilon }=A:\varepsilon$$

From the mechanical point of view, this lack of coincidence between the effective and the nominal strain tensor is essential in representing a fundamental feature of orthotropic damage models, i.e., the fact that the nominal Poisson’s ratio does not remain constant throughout the damage process. Due to the strain equivalence assumption, this feature is not taken into account by the original model.

Exploiting Relations (1) and (13), the effective stress tensor, expressed as a function of the nominal strain ***ε***, is:

(14) $$\bar{\sigma }=D_{0}:\bar{\varepsilon }=D_{0}:A:\varepsilon$$

For the damage model described in [1], the operator ***A*** relating the nominal and the effective stress is the one expressed in Equation (9). Herein, some minor modifications with respect to (9) are introduced for the definition of this fourth-order tensor, even though its fundamental dependence on the damage variables and on a spectral projection operator is maintained. First, the integrity quantities in tension and in compression (1 − *d^+^*) and (1 − *d^−^*) are replaced by their square roots, in order to keep comparable the amount of stiffness degradation between the original model and the new proposal. Secondly, the quantity on which the decomposition is performed is no longer the effective stress, as described in Section 2.1 (see Equations (4) and (5)): the reason lies in the dependence of $\bar{\sigma }$ obtained in an energy-equivalence framework (14) on the fourth-order tensor ***A*** (hence, on the projection operator and on the damage variables), which would make the procedure of the definition of the projection operator iterative. Consequently, the nominal strain tensor ***ε*** is chosen as the variable object of the spectral decomposition, similarly to what is done in [7]; in this way, the algorithmic efficiency related to a strain-based formulation, one of the attractiveness of the original model, is kept unchanged. In addition, a definition for the projection operator slightly different from (5) is here preferred. Specifically, a tensor first introduced in [33] and then presented again in [34] is adopted; its expression is:

(15) $$Q_{CW}=\sum_{\begin{array}{l} i=1 \end{array}}^{3}H\left(\varepsilon_{i}\right)p_{i}⊗p_{i}⊗p_{i}⊗p_{i}+\sum_{\begin{array}{l} i,j=1\\ j>i \end{array}}^{3}\left(H\left(\varepsilon_{i}\right)+H\left(\varepsilon_{j}\right)\right)P^{ij}⊗P^{ij}$$

(16) $$P^{ij}=P^{ji}=\frac{1}{2}\left(p_{i}⊗p_{j}+p_{j}⊗p_{i}\right)$$

where *ε_i_* and ***p_i_*** are the *i*-th principal value and the eigenvector associated with the *i*-th principal direction of the nominal strain tensor ***ε***. *H*(*ε_i_*) is the Heaviside function, such that, if *ε_i_* is positive, *H*(*ε_i_*) = 1; else, *H*(*ε_i_*) = 0. This projection operator does not alter the positive and negative components extracted from the strain tensor, which are exactly the same obtained adopting the conventional ***Q*** (5). The advantage of using it lies in the fact that, when all of the strain eigenvalues *ε_i_* are of the same sign, ***Q_CW_*** satisfies the so-called natural property. For a generic fourth-order projection operator ***P*** that performs a spectral decomposition on a second-order tensor ***a***, this property can be written in the following way:

(17) $$\{\begin{array}{c} P^{+}=I\;\;\begin{array}{c} iff \end{array}\;a_{i}\geq 0\;\begin{array}{c} \left(\forall i=1,2,3\right) \end{array}\\ P^{-}=I\;\;\begin{array}{c} iff \end{array}\;a_{i}<0\;\begin{array}{c} \left(\forall i=1,2,3\right) \end{array} \end{array}$$

The satisfaction of Property (17) is discussed in [11,33].

Referring to Equation (9) and applying the mentioned minor modifications, the proposal A^*^ for the fourth-order tensor ***A***, appearing in Equations (13) and (14), is:

(18) $$A^{*}=\sqrt{1-d^{+}}Q_{CW}+\sqrt{1-d^{-}}\left(I-Q_{CW}\right)$$

In view of this definition, the relations (2), (13) and (14) between the nominal and the effective quantities in an energy-equivalent framework can be re-written as:

(19) $$\sigma =A^{*}:\bar{\sigma }$$

(20) $$\bar{\varepsilon }=A^{*}:\varepsilon$$

(21) $$\bar{\sigma }=D_{0}:\bar{\varepsilon }=D_{0}:A^{*}:\varepsilon$$

Making use of Relations (20) and (21), the equality between the strain energy in the effective and in the real configuration is:

(22) $$\frac{1}{2}\bar{\sigma }:\bar{\varepsilon }=\frac{1}{2}\varepsilon :A^{*}:D_{0}:A^{*}:\varepsilon =\frac{1}{2}\varepsilon :D_{E}:\varepsilon$$

From Equation (22), the form of the secant stiffness fourth-order tensor ***D_E_*** (subscript “*_E_*” stands for energy equivalence) is derived:

(23) $$D_{E}=A^{*}:D_{0}:A^{*}$$

By replacing the definition of A^*^ (18) in Equation (23), the complete expression for the secant matrix ***D_E_*** is:

(24) $$D_{E}=\left[\sqrt{1-d^{+}}Q_{CW}+\sqrt{1-d^{-}}\left(I-Q_{CW}\right)\right]:D_{0}:\left[\sqrt{1-d^{+}}Q_{CW}+\sqrt{1-d^{-}}\left(I-Q_{CW}\right)\right]$$

A similar secant stiffness operator, obtained under the same assumption of energy equivalence, can be found in [14]: there, the projection operator is adopted in its classical form (analogously to Equation (5)), and the split is performed on the nominal stress tensor ***σ***, which, as mentioned earlier, makes iterative the definition of the projection operator.

From Equation (24), some immediate considerations can be given. Firstly, in the absence of damage, the linear elastic stiffness tensor ***D*_0_** is recovered. Secondly, the versatility of the original model in treating the damage-induced anisotropy (see Remark 2.1) is preserved in the new formulation. As a matter of fact, in the case of *ε_i_* with concordant sign (*ε* = *ε**^+^*** or *ε* = *ε**^−^***), an isotropic damage model is regained, while in the case of *ε_i_* with discordant sign, the damaged material is orthotropic, and the coaxiality between the directions of induced orthotropy and the principal directions of the strain and stress tensors is assured. In addition, Equation (23) shows how, due to the major symmetry of the tensor A^*^, the hypothesis of energy equivalence (22) induces automatically major symmetry in the secant stiffness tensor ***D_E_***.

The matrix form of the secant stiffness operator ***D_E_*** is given in (25) in the principal reference system of orthotropy of the damaged material. As done for the expression of the matrix form of ***D_s_*_2_** (11), a 3D strain state composed of two elongation directions and a contraction one is considered:

(25) $$\left[\begin{array}{cccccc} \left(1\;-d^{+})(2G+\lambda \right) & {\left(1\;-d^{-}\right)}^{0.5}{\left(1\;-d^{+}\right)}^{0.5}\lambda & \left(1\;-d^{+}\right)\lambda & 0 & 0 & 0\\ {\left(1\;-d^{-}\right)}^{0.5}{\left(1\;-d^{+}\right)}^{0.5}\lambda & \left(1\;-d^{-}\right)\left(2G+\lambda \right) & {\left(1\;-d^{-}\right)}^{0.5}{\left(1\;-d^{+}\right)}^{0.5}\lambda & 0 & 0 & 0\\ \left(1\;-d^{+}\right)\lambda & {\left(1\;-d^{-}\right)}^{0.5}{\left(1\;-d^{+}\right)}^{0.5}\lambda & \left(1\;-d^{+}\right)\left(2G+\lambda \right) & 0 & 0 & 0\\ 0 & 0 & 0 & {\left[{\left(1\;-d^{-}\right)}^{0.5}+{\left(1\;-d^{+}\right)}^{0.5}\right]}^{2}G/4 & 0 & 0\\ 0 & 0 & 0 & 0 & {\left[{\left(1\;-d^{-}\right)}^{0.5}+{\left(1\;-d^{+}\right)}^{0.5}\right]}^{2}G/4 & 0\\ 0 & 0 & 0 & 0 & 0 & \left(1\;-d^{+}\right)G \end{array}\right]$$

First of all, it is interesting to note how the secant stiffness matrix associated with the operator ***D_E_*** here derived fits perfectly within the framework described in [17], based on the hypotheses of energy equivalence and sum-type symmetrization. Differently from a generic orthotropic material, which is characterized by nine independent material properties, the secant stiffness matrix (25) depends on four variables (the two elastic constants *G* and *λ* of the initial isotropic material and the two damage variables *d^+^* and *d^−^*). This aspect is strictly related to one of the particularities of this formulation, i.e., the coaxiality between directions of induced orthotropy and principal directions of nominal strain and stress tensors.

Moreover, considering the shear stiffness terms in matrix (25), it is possible to see that the shear modulus *G* is reduced by the squares of the sum averages of both *d^+^* and *d^−^*. This constitutes a modification with respect to the secant operators ***D_S_***_1_ and ***D_S_***_2_ (see Equation (11)), due to the adoption of the projection operator ***Q_CW_*** (15) instead of ***Q*** (5); the main positive implication deriving from this choice is the recovery not only of the constitutive law, but also of the secant stiffness matrix of an isotropic damage model, when *ε* = *ε**^+^*** or *ε* = *ε**^−^***.

Besides an evident symmetry visible in Equation (25), the stiffness operator ***D_E_*** (24) is also positive definite, and this can be checked, without loss of generality, by applying Sylvester’s criterion to its matrix form (25). In fact, as demonstrated by the relations (26), all of the minors of (25) are always positive, provided that the damage variables range from zero (virgin material) to one (completely damaged material):

(26) $$\begin{array}{l} \Delta_{1}=D_{E11}=\left(1-d^{+}\right)\left(2G+\lambda \right)\geq 0\\ \Delta_{2}=D_{E11}D_{E22}-D_{E12}^{2}=\left(1-d^{+}\right)\left(1-d^{-}\right){\left(2G+\lambda \right)}^{2}-\left(1-d^{+}\right)\left(1-d^{-}\right)\lambda^{2}\geq 0\\ \Delta_{3}=\left(D_{E11}D_{E22}-D_{E12}^{2}\right)D_{E33}-D_{E23}^{2}D_{E11}+2D_{E23}D_{E12}D_{E13}-D_{E13}^{2}D_{E22}\\ ={\left(1-d^{+}\right)}^{2}\left(1-d^{-}\right){\left(2G+\lambda \right)}^{3}-{\left(1-d^{+}\right)}^{2}\left(1-d^{-}\right)\left(2G+\lambda \right)\lambda^{2}-{\left(1-d^{+}\right)}^{2}\left(1-d^{-}\right)\left(2G+\lambda \right)\lambda^{2}+\\ +2{\left(1-d^{+}\right)}^{2}\left(1-d^{-}\right)\lambda^{3}-{\left(1-d^{+}\right)}^{2}\left(1-d^{-}\right)\left(2G+\lambda \right)\lambda^{2}\geq 0\\ \Delta_{4}=\Delta_{3}D_{E44}=\Delta_{3}G{\left(0.5\left(1-d^{+}\right)+0.5\left(1-d^{-}\right)\right)}^{2}/4\geq 0\\ \Delta_{5}=\Delta_{3}D_{E44}D_{E55}=\Delta_{3}G^{2}{\left(0.5\left(1-d^{+}\right)+0.5\left(1-d^{-}\right)\right)}^{4}/16\geq 0\\ \Delta_{6}=\Delta_{3}D_{E44}D_{E55}D_{E66}=\Delta_{3}G^{3}\left(1-d^{+}\right){\left(0.5\left(1-d^{+}\right)+0.5\left(1-d^{-}\right)\right)}^{4}/16\geq 0 \end{array}$$

Therefore, the new *d^+^*/*d^−^* damage model, based on the hypothesis of energy equivalence, is governed by the secant stiffness operator ***D_E_*** (24), which is symmetric and positive definite; consequently, as further commented in Remark 3.1, it provides an adequate representation of the damage-induced orthotropy.

**Figure 1:**
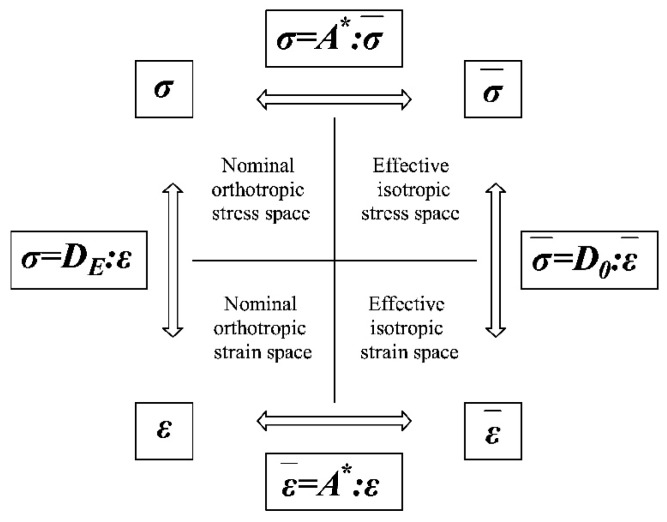
Energy equivalence hypothesis: relations between effective and nominal spaces.

**Remark** **3.1.** *The relationships between the effective and the nominal configuration deriving from the energy equivalence assumption, together with the constitutive laws proper of each space, are summarized in Figure 1*.

An interesting parallelism can be found between these relationships, adopted to derive an orthotropic d^+^/d^−^ damage model, and the mapping procedure presented in [35] to define a damage model for orthotropic materials. In [35], the stress **σ** and strain **ε** tensors of the orthotropic real space are related by means of suitable fourth-order symmetric transformation tensors to those (σ^*^ and ε^*^, respectively) of an equivalent isotropic *solid:*

(27) $$\sigma ={\left(A^{\sigma }\right)}^{-1}:\sigma^{*}$$

(28) $$\varepsilon^{*}=A^{\varepsilon }:\varepsilon$$

*Considering the equivalence between the orthotropic real space and the real damaged material (nominal **σ** and nominal **ε**) and between the mapped isotropic space and the effective undamaged configuration (*$\bar{\sigma }$ *and* $\bar{\varepsilon }$), the similarity of Equations (27) and (28) with (19) and (20) is evident. Specifically, in accordance with [17], in the present orthotropic damage model, the tensor (**A^σ^**)^−**1**^ and **A^ε^** are coincident and equal to the mapping operator A^*^ (18). All of the features required for the mapping operators (**A^σ^**)^−1^ and $A^{\varepsilon }$ (fourth-order tensors, minor and major symmetry, positive definiteness in order to be invertible) are satisfied by A^*^. This observation confirms the effectiveness of the energy-equivalence assumption in representing the damage-induced orthotropy.

### 3.2. Thermo-Dynamic Framework

The free energy potential of the orthotropic damage model presented in Section 3.1 is here provided, in accordance with the energy equivalence assumption (22):

(29) $$\psi \left(\varepsilon ,d^{+},d^{-}\right)=\frac{1}{2}\varepsilon :D_{E}:\varepsilon$$

where the positive definiteness of the secant stiffness fourth-order tensor ***D_E_*** (24) has been proven in Section 3.1.

In order to assess the thermodynamic consistency of the proposed model, the satisfaction of both the first and the second law of thermodynamics needs to be investigated.

On the one hand, the first law of thermodynamics for elastic-degrading materials demands considering the conservation of energy in the unloading-reloading regime, for a fixed state of degradation. As pointed out in [33,34], under those conditions and when non-proportional loadings are applied, damage models including micro-crack closure reopening (MCR) effects may suffer the problem of energy generation/dissipation under closed-load cycles. Specifically, only in the presence of anisotropic degradation, a lack of energy dissipation occurs.

The here-formulated damage model is orthotropic in the sense that a directional degradation in stiffness is induced in the material after surpassing the linear threshold. From the definition of A^*^(18), which, according to Remark 3.1, is the fourth-order operator performing the mapping between the isotropic and the orthotropic spaces, it is asserted that the projection operator ***Q_CW_*** is responsible for the damage-induced orthotropy, since the damage variables are scalars. Both in [33] and in [34], the term “anisotropic degradation” is adopted for describing those damage models that include an anisotropic (or orthotropic) stiffness reduction, beyond the application of the projection operator ***Q***. Therefore, the here-presented damage model cannot be classified as anisotropic according to these references, even if an orthotropic behavior is induced by the damage process; consequently, it observes the first law of thermodynamics. 

On the other hand, the second law of thermodynamics states that the total entropy of an isolated system tends to increase over time, taking into account the irreversibility of the natural processes. This condition can be expressed by the Clausius–Duhem inequality (see [36]):

(30) $$\dot{\gamma }=-\dot{\psi }+\sigma :\dot{\varepsilon }\geq 0$$

Substituting in Equation (30) the rate of the total potential energy expressed in Equation (29), the positiveness of the energy damage dissipation can be rewritten in this way:

(31) $$\dot{\gamma }=\left(\sigma -\frac{\partial \psi }{\partial \varepsilon }\right):\dot{\varepsilon }-\frac{\partial \psi }{\partial d^{+}}\cdot {\dot{d}}^{+}-\frac{\partial \psi }{\partial d^{-}}\cdot {\dot{d}}^{-}\geq 0$$

Since ***ε*** is a free variable, in order to have non-negativeness of $\dot{\gamma }$, the term between round brackets in Equation (31) has to be null, and the constitutive law is established, in accordance with Coleman’s relation ***σ*** = ∂*ψ*/∂***ε*** (see [37]). Consequently, Equation (31) reduces to:

(32) $$\dot{\gamma }=-\frac{\partial \psi }{\partial d^{+}}\cdot {\dot{d}}^{+}-\frac{\partial \psi }{\partial d^{-}}\cdot {\dot{d}}^{-}\geq 0$$

where the partial derivatives of the potential with respect to *d^+^* and *d^−^*, with signs reversed, represent the elastic strain energy release rates produced by a unit growth of the corresponding damage variable; they play the role of thermodynamic forces conjugated to the damage variables. For the sake of brevity, their expressions, together with the discussion of the satisfaction of the second principle of thermodynamics, are provided in Appendix A.

Considering Coleman’s relation ***σ*** = ∂*ψ*/∂***ε*** and the expression of the free energy potential (29), the constitutive law between the nominal stress and strain tensors results, as expected:

(33) $$\sigma =D_{E}:\varepsilon$$

### 3.3. Damage Criterion for Cohesive-Frictional Materials and Damage Evolution Laws

In accordance with the original *d^+^*/*d^−^* damage model, two equivalent stress variables and two independent evolution laws are adopted to define the degradation process in tension and in compression, respectively.

In order to identify the situations of loading, unloading or re-loading, the Kuhn–Tucker relations and the persistency conditions are taken into account, expressed in terms of the equivalent stress variables $\tau^{\pm }$, monitoring the behavior in tension and compression, and of the internal state variables $r^{\pm }$, representing the damage thresholds.

(34) $${\dot{r}}^{\pm }\geq 0$$

(35) $$g^{\pm }=\tau^{\pm }-r^{\pm }\leq 0$$

(36) $${\dot{r}}^{\pm }\cdot g^{\pm }=0$$

(37) $${\dot{r}}^{\pm }\cdot {\dot{g}}^{\pm }=0$$

Combining Equations (34)–(36), it can be asserted that, during the unloading or in the undamaged initial state, $g^{\pm }$ < 0 and ${\dot{r}}^{\pm }=0$, while, in the case of loading, $g^{\pm }$ = 0 and ${\dot{r}}^{\pm }>0$. Moreover, the satisfaction of Equation (37) in the case of loading (${\dot{r}}^{\pm }\geq 0$) allows one to derive the following expression for the internal state variables $r^{\pm }$:

(38) $$r^{\pm }=\max\left(r_{0}^{\pm };\underset{\left[0,t\right]}{\max\left(\tau^{\pm }\right)}\right)$$

where:

(39) $$r_{0}^{\pm }=f_{e}^{\pm }=\gamma_{e}^{\pm }f^{\pm }$$

As shown in Equation (39), the quantities *f_e_^+^* and *f_e_^−^* are related to the corresponding uniaxial peak strengths *f^+^* and *f^−^* by means of the proportional parameters *γ_e_^+^* and *γ_e_^−^*, and they identify the onset of damage in uniaxial tension and compression, respectively.

As can be inferred from Equation (35), the definition of the damage criterion is strictly related to the choice of the equivalent stress variables *τ^+^* and *τ^−^* since the damage limit surface *g*^±^ = 0 is obtained by equating *τ^+^* and *τ^−^* to *r^+^* and *r^−^*, respectively. In this regard, similarly to the choice done in [22,38], the following equivalent stress variables *τ^+^* and *τ^−^* are here considered, inspired by the failure criterion proposed in [10] for the modelling of concrete:

(40) $$\tau^{+}=H\left(\sigma_{emax}\right)\frac{1}{1-\alpha }\frac{f_{e}^{+}}{f_{e}^{-}}\left(\sqrt{3J_{2}}+\alpha I_{1}+\beta \langle \sigma_{emax}\rangle \right)$$

(41) $$\tau^{-}=H\left(-\sigma_{emin}\right)\frac{1}{1-\alpha }\left(\sqrt{3J_{2}}+\alpha I_{1}+\beta \langle \sigma_{emax}\rangle \right)$$

with the material parameters:

(42) $$\alpha =\frac{(f_{b}^{-}/f^{-})-1}{2(f_{b}^{-}/f^{-})-1}\;\beta =\left(1-\alpha \right)\frac{f_{e}^{-}}{f_{e}^{+}}-\left(1+\alpha \right)$$

In Expressions (40)–(42), *f_b_^−^* is the biaxial compressive strength of the material, while the first invariant $I_{1}$, the deviatoric second invariant $J_{2}$, the maximum (*σ_emax_*) and minimum (*σ_emin_*) principal stress values are referred to the elastic stress tensor ***σ_e_***, whose definition is:

(43) $$\sigma_{e}=D_{0}:\varepsilon$$

The choice of ***σ_e_***, as the quantity determining the equivalent variables, is addressed to avoid an iterative procedure for the computation of *d^+^* and *d^−^*, which would be otherwise necessary opting for the effective stress $\bar{\sigma }$ defined in (21).

The parameters introduced in Equation (42) are related to the frictional properties of the material, while *r*_0_*^−^*, i.e., the compressive uniaxial strength at the onset of damage (see Equation (39)), represents the cohesive contribution in the undamaged state.

**Figure 2:**
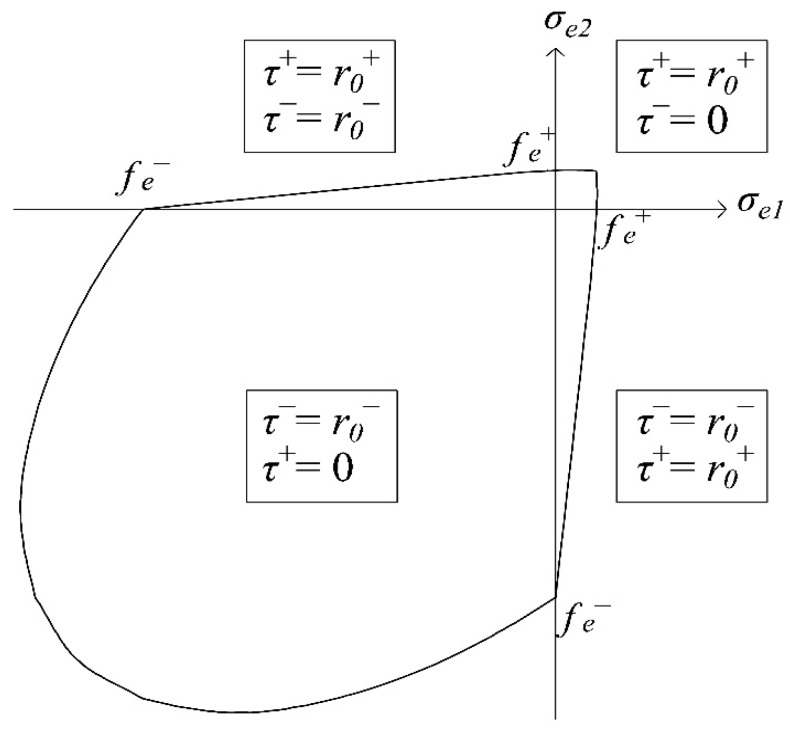
Damage surface in plane stress conditions, for *α* = 0.121 and *β* = 7.667.

The limit surface for the activation of damage in the initial elastic stage, derived by equating *τ^+^* and *τ^−^* (Equations (40) and (41)) to *r*_0_*^+^* and *r*_0_*^−^* (Equation (39)), is plotted in Figure 2 in the elastic stress principal space, for the plane stress case. In the first and third quadrants, thanks to the presence of the Heaviside functions in Equations (40) and (41), the limit surface is ruled by *τ^+^* and *τ^−^*, separately, meaning that only tensile damage can be activated in the first quadrant and only compressive damage in the third one. Specifically, in biaxial compression, the Drucker–Prager criterion is recovered. Differently, in the second and fourth quadrants, tensile and compressive damages are activated contemporarily because of the perfect overlapping between the surfaces identified by *τ^+^* and *τ^−^*. Such a modelling of the failure conditions in the second and fourth quadrants is a betterment with respect to the damage limit surface considered in the original model; as a matter of fact, in [1], the behavior of the material in these quadrants is not represented directly, but is only considered as the intersection of the two distinct failure criteria in pure tension (first quadrant) and pure compression (third quadrant).

In this work, the damage evolution laws for *d^+^* and *d^−^* are explicitly defined as monotonically-increasing functions of the corresponding thresholds *r^+^* and *r^−^*, such that ${\dot{d}}^{\pm }\geq 0$. Specifically, the formulation proposed in [39] is herein considered, which models both the strain-hardening and the strain-softening in the uniaxial stress-strain law, adopting a parabolic and an exponential trend, respectively:

(44) $$d^{\pm }\left(r^{\pm }\right)=A_{d}^{\pm }\frac{f^{\pm }}{r^{\pm }}{\left(\frac{r^{\pm }-r_{0}^{\pm }}{f_{p}^{\pm }-r_{0}^{\pm }}\right)}^{2}\;\begin{array}{c} \begin{array}{c} \begin{array}{c} r_{0}^{\pm }\leq \end{array} \end{array} \end{array}r^{\pm }\leq f_{p}^{\pm }$$

(45) $$d^{\pm }\left(r^{\pm }\right)=1-\frac{f^{\pm }}{r^{\pm }}\cdot \exp\left(2H_{d}^{\pm }\cdot \left(\frac{f_{p}^{\pm }-r^{\pm }}{f^{\pm }}\right)\right)\;\begin{array}{c} r^{\pm }>f_{p}^{\pm } \end{array}$$

Two further variables, *f_p_^+^* and *f_p_^−^*, appearing in Equations (44) and (45), have to be introduced: they represent the damage limit surfaces at the corresponding peak strengths and they depend on *f^+^* and *f^−^* by means of the proportional factors *γ_p_^+^* and *γ_p_^−^* (*γ_p_^±^* ≥ 1):

(46) $$f_{p}^{\pm }=\gamma_{p}^{\pm }f^{\pm }$$

Moreover, the definition for the positive parameters *A_d_* and *H_d_* is needed:

(47) $$A_{d}^{\pm }=\frac{f_{p}^{\pm }-f^{\pm }}{f^{\pm }}\;\frac{1}{H_{d}^{\pm }}=2\left(\frac{EG_{f}^{\pm }}{{\left(f^{\pm }\right)}^{2}}\frac{1}{l_{dis}}-\frac{1}{2}\frac{f_{p}^{\pm }}{f^{\pm }}-{\bar{A}}_{d}^{\pm }\right)$$

with ${\bar{A}}_{d}^{\pm }=A_{d}^{\pm }\left({\left(f_{p}^{\pm }\right)}^{3}-3f_{p}^{\pm }\cdot f_{e}^{\pm 2}+2\cdot f_{e}^{\pm 3}\right)/\left(6f^{\pm }{\left(f_{p}^{\pm }-f_{e}^{\pm }\right)}^{2}\right)$.

The dependence of the exponential strain softening laws (45) on the fracture energies in tension and compression, *G_f_^+^* and *G_f_^−^*, and on the length related to the discretization, *l_dis_*, is introduced in order to ensure mesh-size objective results, in accordance with the crack-band theory presented in [40]. The definition of the crack width parameter *l_dis_* can be done taking into account [41] and is related to the area (volume) of the finite elements in 2D (3D) problems.

**Figure 3:**
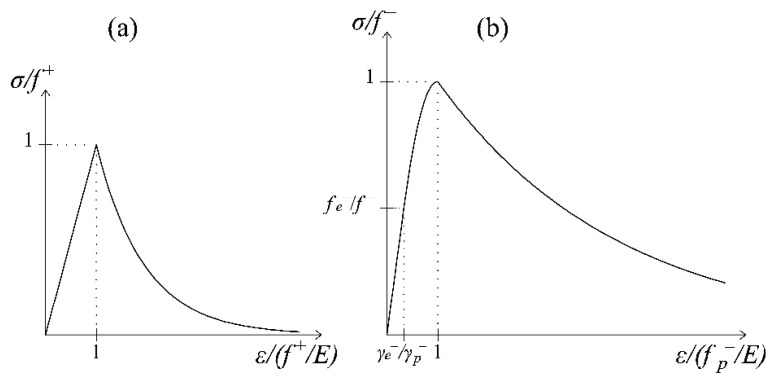
Uniaxial normalized *σ-ε* curves: (**a**) softening behavior in tension and (**b**) in compression.

For cohesive-frictional materials, which are the main objective of the present study, it is realistic to consider the parabolic hardening before strain softening only for the compressive variable *d^−^*, i.e., to consider *f_p_^−^* > *f^−−^* > *f_e_^−^*. For the tensile damage variable *d^+^*, the hypothesis of *f_p_^+^ = f^+^ = f_e_^+^* is made, meaning that the onset of damage is immediately followed by a strain softening behavior. The uniaxial stress strain curves resulting from these assumptions under tension and under compression are shown in Figure 3a,b, respectively.

### 3.4. Algorithm for the d^+^/d^−^ Damage Model

To have further insight into the *d^+^*/*d^−^* damage model proposed in this section, the main points of its numerical algorithm are summarized in Table 1. 

Due to the material non-linearity, an incremental-iterative procedure is required. Here, the details about the iterative process to assure equilibrium are not provided, and only the numerical scheme for the derivation of the local constitutive equation is shown. The adoption of a displacement-based finite element method allows one to determine the nominal strain tensor ***ε*** at the beginning of each loading step (or at the beginning of every equilibrium iteration) and consequently allows the explicit computation of damage from the strains.

## 4. Multidirectional *d^+^*/*d^−^* Damage Model for Cyclic Loadings

### 4.1. Limitations of the Original d^+^/d^−^ Formulation

Together with the damage-induced anisotropy, another essential feature that needs to be taken into account in the constitutive behavior of cohesive frictional materials as concrete is the modelling of the microcrack closure-reopening (MCR) effects [42]. These effects consist of the partial or total recovery of the material stiffness upon load reversal, related to the closure of previously-generated microcracks. As shown in [1], the original *d^+^*/*d^−^* damage formulation is able to capture this unilateral behavior in the presence of a 1D tension-compression cyclic load history. This is achieved thanks to the spectral decomposition of the effective stress tensor (4), which allows one to consider the axial stiffness first affected by *d^+^* in tension and then unaffected (or affected by *d^−^*) in compression. Likewise, the new damage model proposed in Section 3 ensures such a unilateral effect upon 1D loading reversal, adopting the strain decomposition projection operator (15). 

However, neither with the original formulation nor with the new damage model, the microcrack closure-reopening effects in the presence of a generic cyclic load history (not necessarily uniaxial) can be adequately taken into account. 

An illustrative case is for instance a problem involving pure shear cyclic loading conditions, characterized by a swapping between minimum and maximum principal strain directions in correspondence of the loading reversal. At the end of the first loading path, both the damages in tension and in compression are assumed different from zero and equal to *d*_1_*^+^* and *d*_1_*^−^*; after loading reversal, *d*_1_*^+^* is assigned to the current maximum principal direction, although this direction has not been affected by tensile damage in the previous loading stage. The same happens for *d*_1_*^−^*, and consequently the material response does not show any stiffness recovery associated with the crack closure phenomena. 

From this example, the limitation of the model due to the scalar representation of damage, which implies the impossibility of maintaining permanent memory of the damage orientation, is evident. Non-negligible consequences of this fact can be observed in cyclic loadings and, in a more general framework, for non-proportional loadings, since those cases are characterized by a rotation of the minimum and maximum strain/stress principal directions. 

### 4.2. Multidirectional Procedure

Without the necessity of resorting to a tensor definition for damage, a procedure able to enhance the stiffness recovery capabilities of the *d^+^*/*d^−^* model is here presented. The interest is specifically addressed to the case of cyclic loadings, either considered alone or preceded and/or followed by non-cyclic (permanent or proportionally increasing) loadings. The essential aspects of this approach lie in maintaining only two scalar variables in the constitutive law (33) and, contemporarily, in preserving the memory of the directionality of the damage process. The former aspect is related to the will of not altering the basic scheme of the original formulation, which has proven to be effective and adequate in modelling the material behavior of concrete and concrete-like materials; as discussed in Section 4.1, the latter is instead a fundamental requirement in order to capture the MCR effects in the presence of cyclic loadings. 

Although the procedure holds general validity, for the sake of clarity, it is described in the follow-up with reference to plane problems. 

The main novelty with respect to the standard computation of the *d^+^* and *d^−^* damage variables (as described in Section 3.3) is represented by the possible activation of a “multidirectional” damage model. The concept of a “multidirectional” damage model refers to a partition of the plane into two regions for tensile damage, each one endowed with its own *d^+^* and *r^+^* values, and into two regions for the compressive one, each one endowed with its own *d^−^* and *r^−^* values. Hence, all of the directions contained in a region are associated with its own *d^+^* or *d^−^* degradation parameters. A tensile (compressive) damage value is associated with a certain region depending on the maximum (minimum) principal strain direction that has generated it. Between the two values of *d^+^* (*d^−^*), the active one, i.e., the one adopted in the constitutive law (33), is computed starting from the *d^+^* (*d^−^*) already developed in the region, which includes the current maximum (minimum) principal strain direction. The other values of damage are instead maintained temporarily inactive with the possibility of being re-activated when the rotation of the maximum/minimum principal directions occurs. This means that, with the activation of a multidirectional *d^+^*/*d^−^* damage model, two independent damage evolution laws for tension and two independent damage evolution laws for compression are considered, differing for the directions in which they act.

In this regard, an important remark is in order: the switch from a region to another one does not alter the irreversibility of the damage process; in fact, within each region, the damage variables are monotonically increasing functions, and their updating follows the Kuhn–Tucker and persistency conditions (34)–(37) and the evolution laws (44) and (45).

It is useful to examine separately two types of cyclic conditions: (i) cyclic loading characterized by a fixed principal reference system and with only swapping between maximum and minimum (or vice versa) principal directions; (ii) cyclic loading with continuous rotation of maximum and minimum principal directions. This distinction is mainly justified by the fact that, as discussed in the follow-up, different stiffness recovery capabilities are expected and, consequently, have to be modelled, in the two cases.

**Figure 4:**
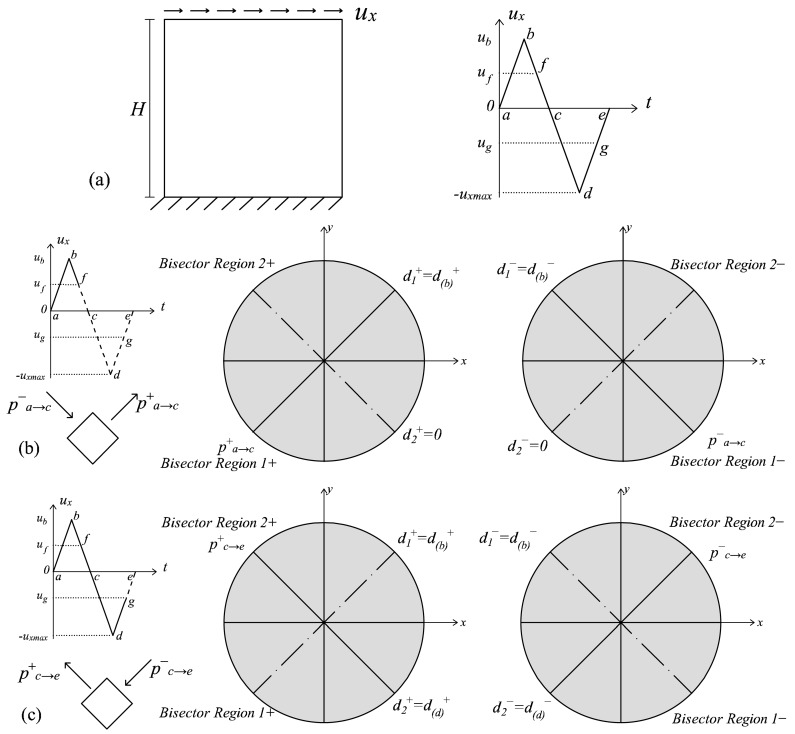
Multidirectional damage procedure in the case of cyclic loading of Type (i): (**a**) problem statement; (**b**) identification of the damage regions and damage variables before the load reversal and (**c**) after the load reversal.

For loading Type (i), the cyclic conditions imply a π/2 rotation of the principal directions in the occurrence of swapping. This orthogonality allows one to assume that the full fracture energies *G_f_^+^* and *G_f_^−^* are consumed in each principal direction independently of one another. From the modelling point of view, this translates into considering active the multidirectional damage procedure from the beginning of the cyclic loading history, with the consequence that a complete regain of the initial stiffness is assured when, for the first time, a swapping between the maximum and minimum (or vice versa) principal directions occurs. Since the principal reference system is fixed in these loading conditions, also the damage regions in which the space is partitioned are considered fixed: their bisectors are assumed coincident with the principal directions, and their amplitude is equal to π/2. The problem described in Figure 4a, characterized by a panel subjected to a cyclic horizontal displacement *u_x_* inducing shear, is an example of these cyclic loading histories. In Figure 4b,c, the working principles of the multidirectional damage model are shown: two damage values for tension and two damage values for compression are kept in memory based on the current principal strain directions (which are ***p^+^***_a→c_, ***p^−^***_a→c_ in Figure 4b and ***p^+^***_c→e_, ***p^−^***_c→e_, in Figure 4c). A perfect overlapping between the damage regions in tension and the damage regions in compression, which are fixed throughout the cyclic loading history, is visible. 

For loading Type (ii), the assumption of considering from the beginning two independent damage evolution laws in tension and in compression does not hold anymore, since, instead of the brusque π/2 rotation of the principal directions typical of a loading Type (i), there is a continuous rotation of the principal reference system. Therefore, an estimation during the loading history of the deviation of the principal directions with respect to the initial conditions becomes necessary in order to evaluate the possible activation of a multidirectional damage procedure. In this regard, two sets of variables are introduced. The first one, the equivalent deviation quantity *τ^+^_θ_* (*τ^−^_θ_*), is defined in the following way:

(48) $$\tau_{\theta }^{\pm }=\cos\left(\theta_{\tau }^{\pm }\right)$$

where *θ^+^_τ_* (*θ^−^_τ_*) is the absolute value of the angle between the current maximum (minimum) principal strain direction and the maximum (minimum) principal strain direction at the initial stage of the cyclic loading history; it ranges from zero to π/2. The second quantity is the internal state variable *r*^±^*_θ_*, which assumes the role of a threshold angle; moreover, it allows one to identify the bisectors of the two regions with distinct *d^+^* (*d^−^*) damage values. 

To monitor the multidirectional damage model procedure and to identify and update the damage regions in the presence of loading or unloading conditions, the following relations are considered:

(49) $${\dot{r}}_{\theta }^{\pm }\leq 0$$

(50) $$g_{\theta }^{\pm }=\tau_{\theta }^{\pm }-r_{\theta }^{\pm }\geq 0$$

(51) $${\dot{r}}_{\theta }^{\pm }\cdot g_{\theta }^{\pm }=0$$

(52) $${\dot{r}}_{\theta }^{\pm }\cdot {\dot{g}}_{\theta }^{\pm }=0$$

The similarity with the Kuhn–Tucker and persistency conditions (34), (35), (36) and (37) adopted for the onset and evolution of the damage variables is evident.

The definition of *r*^±^*_θ_* is derived from Equation (52) and is equal to:

(53) $$r_{\theta }^{\pm }=\cos\left(\theta_{r}^{\pm }\right)=\min\left(\cos\left(\theta_{min}\right);\underset{\left[0,t\right]}{\min\left(\tau_{\theta }^{\pm }\right)}\right)$$

where *θ_min_* is the minimum deviation for which an independent treatment of the damage variables depending on the spatial orientation is valid. As *r^+^* (*r^−^*) (Equation (38)) allows one to define the evolution of the damage variable *d^+^* (*d^−^*) (see Equations (44) and (45)), *r^+^_θ_* (*r^−^_θ_*) allows one to identify the evolution of the bisectors and of the amplitude of the two regions with distinct *d^+^* (*d^−^*) values. Specifically:

(54) $$\begin{array}{cccc} bisector_{1,2}^{\pm }=\pm \theta_{r}^{\pm } & if & r_{\theta }^{\pm }\geq \cos\left(\pi /4\right) & \theta_{r}^{\pm }\leq \pi /4 \end{array}\;\begin{array}{cccc} bisector_{1,2}^{\pm }=\pm \pi /4 & if & r_{\theta }^{\pm }<\cos\left(\pi /4\right) & \theta_{r}^{\pm }>\pi /4 \end{array}$$

where *bisector*^+^_1,2_ and *bisector*^−^_1,2_ refer to the directions with respect to the initial maximum and minimum principal strain directions, respectively. The amplitude of each region is exactly equal to the double of the angle generated between the bisectors and the initial directions. Due to the orthogonality between maximum and minimum principal directions, the equality between *r^+^_θ_* and *r^−^_θ_* is always assured; this implies that the activation of the bidirectional procedure and the updating of the damage regions in tension and compression occur simultaneously. 

If *r*^±^*_θ_* = cos(*θ_min_*) ($g_{\theta }^{\pm }>0$ and ${\dot{r}}_{\theta }^{\pm }=0$), the differentiation of the damage values in tension and compression depending on the principal directions is not performed, which translates in the fact that *d^+^* and *d^−^* are computed as in the original formulation. When *τ*^±^*_θ_* = cos(*θ_min_*) (*g*^±^*_θ_* = 0) for the first time, the bidirectional damage model is activated and the bisectors of the damage regions in tension and compression are the directions inclined by the angles $\pm \theta_{min}$.

On the one hand, in case of loading ($g_{\theta }^{\pm }=0$ and ${\dot{r}}_{\theta }^{\pm }<0$) and when *θ*^±^*_r_* < π/4, the bisectors rotate, coinciding always with the principal directions; their rotation is accompanied by a continuous increase of the regions’ amplitude, which is assumed equal to 2 *∙ θ*^±^*_r_*. In this way, two directions initially belonging to the same region are affected by the same degradation values for the whole damage process. In the case of loading ($g_{\theta }^{\pm }=0$ and ${\dot{r}}_{\theta }^{\pm }<0$) and when *θ*^±^*_r_* > π/4, the rotation of the bisectors stops in order to avoid overlapping between the two tensile (compressive) damage regions; their bisectors are identified by π/4, and their amplitude is equal to π/2. On the other hand, in the case of unloading (*r*^±^*_θ_* < cos(*θ_min_*), $g_{\theta }^{\pm }>0$ and ${\dot{r}}_{\theta }^{\pm }=0$), the damage regions are fixed to the values assumed at the last loading step.

**Figure 5:**
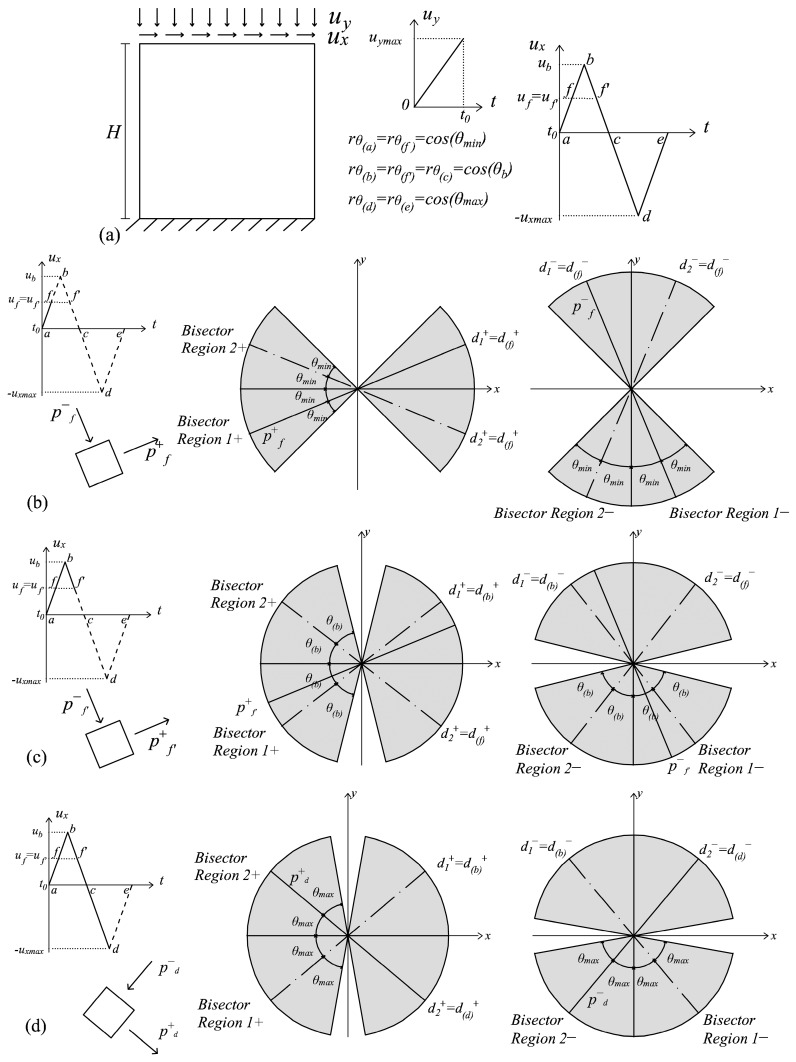
Multidirectional damage procedure in case of cyclic loading of Type (ii): (**a**) problem statement and identification of the damage regions and damage variables in three different situations: (**b**) in correspondence with the activation of the multidirectional procedure; (**c**) in unloading conditions and (**d**) in loading conditions.

Those problems where a proportionally-varying load is applied before a cyclic history are included among the loading conditions of Type (ii), since the simultaneous presence of a permanent load and of a cyclic one is responsible for the rotation of the principal directions. In these specific cases, the equivalent quantities *τ*^±^*_θ_* defined in (48) represent the deviation of the current principal directions with respect to the principal directions of the permanent load. An example of these loading conditions is shown in Figure 5a, where a structural element is subjected first to an increasing vertical contraction and then to a cyclic horizontal displacement *u_x_*. The essentials of the multidirectional damage model in the presence of rotating principal directions are illustrated making reference to this problem. In Figure 5b, the damage values in correspondence with the activation of the multidirectional procedure (point *f* of the loading history) are shown: no distinction between the damage values in the tensile and compressive regions is visible from *a* to *f*, as in the standard *d^+^*/*d^−^* formulation. Moreover, Figure 5c describes the damage distribution in the unloading conditions ($g_{\theta }^{\pm }>0$ and ${\dot{r}}_{\theta }^{\pm }=0$), after the achievement of the peak displacement in *b*. In this case, the damage values and the damage regions are the ones assumed in correspondence with *b*, and the multidirectional procedure is activated since only in the regions including the current principal strain directions (Region 1+ and Region 1−), the damage variables have evolved with respect to the values in *f*. Finally, in Figure 5d, the damage distribution in correspondence with the maximum displacement (point *d* of the loading history) is depicted: due to the loading conditions ($g_{\theta }^{\pm }=0$ and ${\dot{r}}_{\theta }^{\pm }<0$) and the satisfaction of the inequality *θ*^±^*_r_* < π/4, a growth in the regions’ amplitude and a further rotation of the bisectors is visible comparing Figure 5c,d. In all of the situations analyzed in Figure 5, the regions for the tensile and compressive damage never perfectly overlap since the maximum rotation that a principal direction covers, i.e., 2 *∙ θ*^±^*_r_*, is in any case lower than π/2.

In view of this example, the introduction of a minimum threshold *θ_min_* can be better understood: it has the objective of delaying the activation of a multidirectional damage model, and consequently, it implies the possibility of a partial stiffness recovery in a generic loading history. The modelling of a partial stiffness recovery seems adequate for loading conditions of Type (ii): in fact, a continuous rotation of the principal directions without any brusque variation allows one to transfer a certain amount of damage accumulated in a direction to the closest ones.

The two limit cases, characterized by no stiffness recovery and by complete stiffness recovery, are however covered by the present formulation: if the cyclic history does not generate relevant deviations from the principal reference configuration of the permanent load, no stiffness recovery occurs; if no damage is present before the activation of the multidirectional damage procedure, a total stiffness recovery is obtained.

Adequate values for *θ_min_* range from π/12 to π/6, in accordance with the choice done in [43] for defining the threshold angle in a multidirectional fixed crack model. As a matter of fact, a strong analogy between the present formulation and a multidirectional fixed crack model can be found in the common preservation of memory regarding damage orientation and in the common use of a minimum deviation ruling the independent treatment of the cracking phenomenon.

### 4.3. 3D Extension of the Multidirectional Procedure

The 3D extension of the here-described multidirectional damage model has to be further analyzed, but it seems possible by referring to the same concepts introduced for plane problems and adopting the same distinction in cyclic loading Type (i) and cyclic loading Type (ii). Specifically, as in 2D, the partition of the space in regions for tensile and compressive damage is performed, *d^+^* (*d^−^*) is assigned to all of the regions including an eigenvector associated with a positive (negative) principal strain, and the active value of the tensile damage variable (compressive damage variable) is computed considering the current maximum (minimum) principal strain direction. 

As regards loading Type (i), the multidirectional procedure is considered active from the beginning of the cyclic loading history, and the space is divided into three regions for tensile damage and into three regions for compressive damage. As in 2D, the bisectors of the damage regions coincide with the principal directions, which are fixed throughout the loading history, and the amplitude of each region is equal to π/2, meaning that each principal direction that forms an angle *θ* with the bisector of a region, such that |cos(*θ*)| ≥ cos(π/4) belongs to that region. 

For loading Type (ii), characterized by a continuous rotation of the principal directions, the activation of the multidirectional procedure and the updating of the damage regions are ruled by the same conditions holding in 2D, i.e., the conditions in (49)–(52). Likewise, the deviation with respect to the initial configuration is evaluated resorting to Definition (48), and the threshold quantity is provided by (53). Since the active damage values are computed based on the maximum and minimum principal directions (not the intermediate principal directions) and the loading conditions foresee a continuous, and not abrupt, rotation of the principal reference system, additional damage regions associated with the rotation of the intermediate principal direction are not necessary. Therefore, as in 2D problems, two regions for tensile damage, related to the rotation of the maximum principal direction, and two regions for compressive damage, related to the rotation of the minimum principal direction, are considered. If the bisector of the tensile damage Region 1, coinciding with the current maximum principal direction (loading conditions), is inclined by an angle +*θ^+^_r_* (or +π/4) with respect to the initial maximum principal strain direction, the bisector of the tensile damage Region 2 is automatically defined as the direction inclined of an angle −*θ^+^_r_* (or −π/4) with respect to the initial maximum principal strain direction and belonging to the plane in which the maximum principal strain direction has rotated in the loading history. A direction is included in a region if it forms an angle *θ* with the bisector of that region, such that |cos(*θ*)| ≥ |cos(*θ*^±^*_r_*)|.

### 4.4. Algorithms for the Multidirectional d^+^/d^−^ Damage Model

In the present section, the numerical algorithm of the multidirectional damage procedure in a displacement-based finite element framework is described, with reference to plane problems. As done in Section 3.4 for the energy-equivalent *d^+^*/*d^−^* damage model, only the details about the derivation of the local constitutive equation are provided and not the details about the iterative equilibrium procedure. Specifically, the distinction discussed in Section 4.2 between cyclic load Type (i) and cyclic load Type (ii) is here maintained: the former case is contained in Table 2, while the latter in Table 3. Besides the initialization of the quantities at the load increment *n = 0*, the multidirectional damage model requires also the initialization of the bisectors of the damage regions, which is performed in the first load step (*n = 1*).

Three subroutines are adopted in Table 2 and Table 3, where the input parameter *θ* represents one half of the amplitude of the damage regions.

- “*Damage multidirectional saving* (*θ*)” allows one to keep permanent memory of the damage orientation, assigning a tensile (compressive) damage value to a certain region depending on the maximum (minimum) principal strain direction that has generated it;
- “*Damage multidirectional updating* (*θ*)” provides the active damage values and the corresponding active damage thresholds, computed with reference to the current principal strain directions and the current equivalent stress quantities *τ*^±^(Equations (40) and (41));
- “*Bisectors updating* (*θ*)”, useful in the presence of cyclic load Type (ii), modify the bisectors and the amplitudes of the damage regions, according to Condition (54), when $g_{\theta }^{\pm }=0$ and ${\dot{r}}_{\theta }^{\pm }<0$.

## 5. Enhanced Capabilities of the New Multidirectional *d^+^*/*d^−^* Damage Model

To have a further insight into the energy-equivalent symmetric damage model proposed in Section 3 and the multidirectional procedure described in Section 4, some problems are solved at a finite element level and commented on. Specifically, the betterments with respect to the original *d^+^*/*d^−^* damage model presented in Section 2.1 are underlined. In order to focus on the most important aspects, all of the comparisons are performed adopting for both the original and the new formulations the same equivalent stress quantities and the same damage evolution laws, in particular the ones presented in Section 3.4. 

The constitutive properties adopted in the numerical analyses are given in Table 4. They are representative of masonry, once the cohesive-frictional materials more of interest in civil structural applications. The values presented in Table 4 satisfy Inequalities (A7) and (A8), which are the only conditions to be evaluated for the consistent application of the damage model.

### 5.1. Enhanced Representation of the Damage-Induced Orthotropy

As extensively discussed in Section 3.1, the proposed energy-equivalent damage model, based on the consistent secant operator ***D_E_*** (24), represents a step forward with respect to the previous formulation thanks to an adequate consideration of the Poisson effect on the representation of the damage-induced orthotropy. The implications deriving from this fact are shown with reference to the plane stress problem of a bar loaded in tension along the *x*-axis.

**Figure 6:**
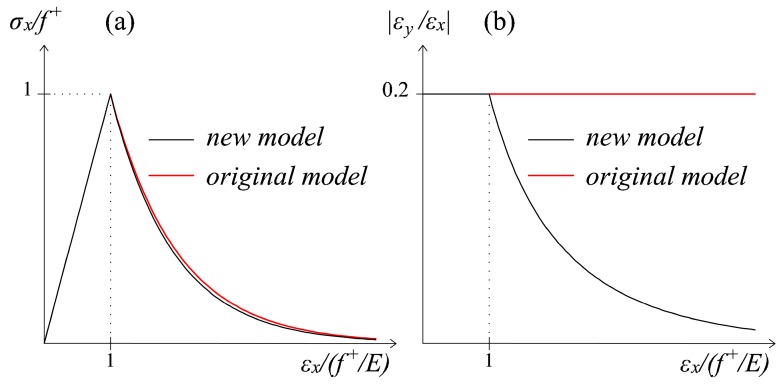
Comparison between the original formulation and the new energy-equivalent model for the problem of a bar uniaxially loaded in tension: (**a**) *σ_x_-ε_x_* curves and (**b**) the nominal Poisson’s ratio trends.

Comparing the normalized *σ_x_ − ε_x_* curves obtained with the original model (Section 2.1) and with the energy-equivalent one (Section 3), no significant differences can be found in the softening response (see Figure 6a). The substantial improvement of the proposed damage model is instead visible looking at the strain behavior in the transversal direction *y* (see Figure 6b). By plotting in abscissa the longitudinal strain *ε_x_* and in ordinate the absolute value of *ε_y_*/*ε_x_*, i.e., the nominal Poisson’s ratio predicted by the models, completely different trends result.

On the one hand, with the original damage model, the nominal Poisson’s ratio maintains constant throughout the loading history, meaning that the transversal contractions *ε_y_* increase unrealistically throughout the whole loading history, together with the growing of the axial elongations *ε_x_*. On the other hand, the adoption of the proposed orthotropic symmetric damage model allows one to take into account a progressive reduction of the nominal Poisson’s ratio with the development of tensile damage. Specifically, by deriving the stiffness matrix ***D_E_*** (24) for a uniaxial tensile plane stress problem and by equating to zero the transversal normal stress *σ_y_*, it is easy to find the expression of the nominal Poisson’s ratio predicted by the model, which is $\bar{\nu }=-\varepsilon_{y}/\varepsilon_{x}=\sqrt{1-d^{+}}\cdot \nu$.

As noticed in [28], the feature of a constant Poisson ratio is usually typical of isotropic damage models, and it is not coherent within the framework of the classical smeared crack models [44,45]. Under a mechanical point of view, a crack generation under uniaxial tension is accompanied by a release of the transversal strains, due to the progressive loss of coupling between longitudinal and transversal directions induced by the degradation process. Thanks to the satisfaction of the symmetry requirement, the enhanced orthotropic damage formulation here proposed is able to simulate adequately such a lateral deformation behavior, whose importance has been generally overlooked by other damage models for quasi-brittle materials (see [46] for a comprehensive discussion about this topic).

### 5.2. Enhanced MCR Effects under Cyclic Loading

In order to show the capabilities of the multidirectional damage model described in Section 4, the qualitative structural responses obtained in presence of Type (i) and Type (ii) cyclic loadings are discussed. 

**Figure 7:**
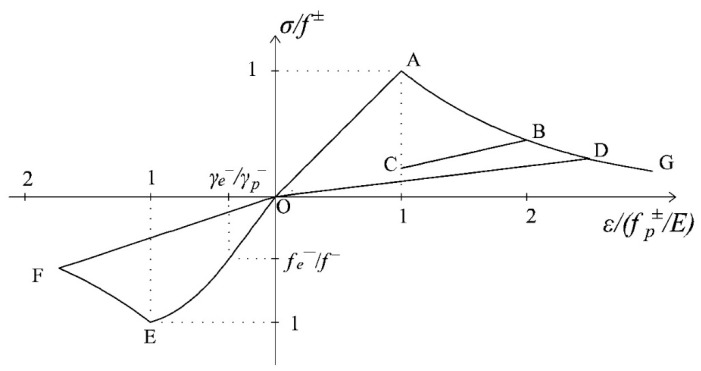
1D cyclic loading history.

First of all, a cyclic uniaxial load history, belonging to the category of loading Type (i), is considered. Since the constitutive behaviors obtained by adopting the original *d^+^*/*d^−^* damage model (Section 2) and the new *d^+^*/*d^−^* model (Section 3) enriched with the multidirectional damage procedure are qualitatively the same in terms of unilateral effects, only one *σ-ε* curve is shown, in Figure 7.

It refers to the following loading sequence: a loading in tension with an exceeding of the initial damage threshold *r*^±^_0_ (O-A-B), a partial unloading followed by a reloading in tension with further progression of damage (B-C-D), an unloading in compression with development of softening (D-O-E-F) and a further reloading in tension (F-O-D-G). It is evident from Figure 7 that the initial stiffness recovery in the transition from tension to compression (D-O-E) is captured. Therefore, in uniaxial loading conditions, the multidirectional damage model coincides with the standard procedure in the modelling of unilateral effects.

**Figure 8:**
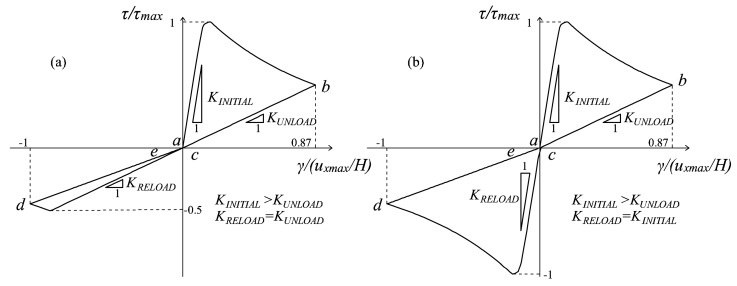
Structural response for the problem represented in Figure 4a: (**a**) damage formulation presented in Section 2 and (**b**) multidirectional damage model.

The advantages of the proposed formulation are demonstrated in more generic loading conditions, as the ones previously commented on and represented in Figure 4a (Type Load (i)) and in Figure 5a (Type Load (ii)). Regarding the problem of the panel subjected to pure shear cyclic loading conditions (Figure 4a), the normalized τ-γ responses obtained with the original formulation and with the multidirectional damage procedure are displayed in Figure 8a,b. In this case, the differences in terms of stiffness recovery are clear: in Figure 8a, no stiffness recovery is visible when the inversion of the horizontal displacement occurs (in the stage from the point *b* of the loading history to *d*), while in Figure 8b, the regain of the initial stiffness is present. As commented on in Section 4.2, the total stiffness recovery is justified by the orthogonality of the tensile and compressive directions between the first loading part (going from the point *a* of the loading history to *c*) and the second loading part (going from *c* to *e*). 

As regards the problem of the panel subjected first to a contraction and then to a shear cyclic loading history (Figure 5a), three combinations of horizontal and vertical displacement values are analyzed, differing for the ratio *m* = |*u_xmax_*/*u_ymax_*| between the maximum *u_x_* and maximum *u_y_* attained. Different values of this ratio translate into different maximum values assumed in the loading history by the variable *r*^±^*_θ_* (Equation (53)). The minimum deviation *θ_min_*, first introduced in Equation (53), is chosen equal to π/8.

**Figure 9:**
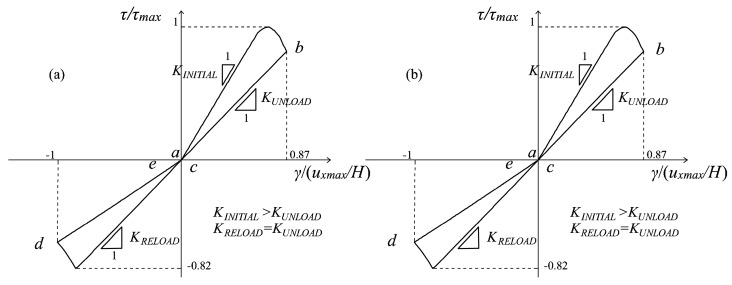
Structural response for the problem represented in Figure 5a, ratio *m* = 1: (**a**) damage formulation presented in Section 2 and (**b**) multidirectional damage model.

On the one hand, in Figure 9, the value *θ^+^_τ_* is lower than *θ_min_*, meaning that the multidirectional procedure is never activated (*m* = 1). As a matter of fact, no differences can be found between the *τ-γ* curves obtained with the original damage formulation (Figure 9a) and with the multidirectional one (Figure 9b), both characterized by the absence of stiffness recovery when the horizontal displacement changes sign. 

In these conditions, the lack of MCR capabilities is adequate because the maximum and minimum principal strain directions responsible for the damage generation do not deviate significantly from the principal configuration induced by the permanent vertical displacement.

**Figure 10:**
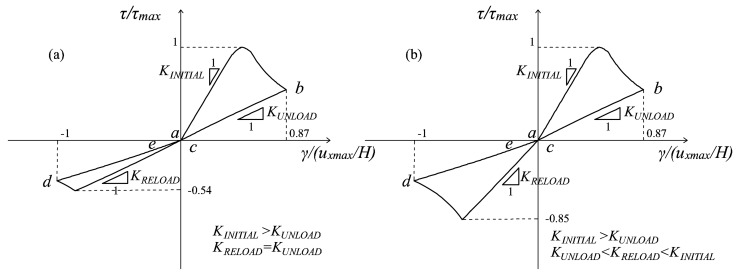
Structural response for the problem represented in Figure 5a, ratio *m* = 1.8: (**a**) damage formulation presented in Section 2 and (**b**) multidirectional damage model.

**Figure 11:**
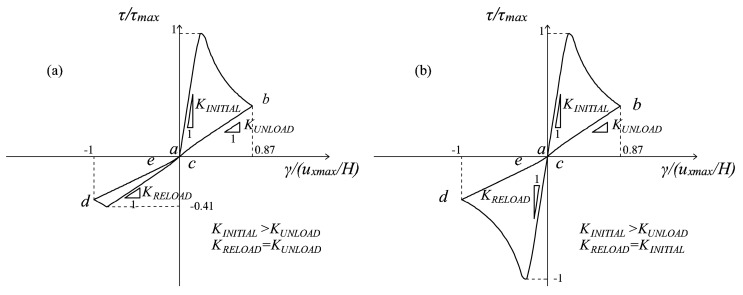
Structural response for the problem represented in Figure 5a, ratio *m* = 8: (**a**) damage formulation presented in Section 2 and (**b**) multidirectional damage model.

On the other hand, in both Figure 10 and Figure 11, the maximum deviation is greater than *θ_min_*, and the ratios between the horizontal and vertical displacements considered are *m* = 1.8 and *m* = 8, respectively. In both of these cases, the enhanced microcrack closure reopening capabilities of the multidirectional damage procedure are evident: the curves resulting from the original damage formulation (Figure 10a and Figure 11a) do not show any stiffness recovery while the multidirectional damage procedure (Figure 10b and Figure 11b) simulates the stiffness regain (from *b* to *d*) satisfactorily. 

Specifically, for *m* = 1.8, the stiffness recovery is only partial (Figure 10b), while for *m* = 8 (Figure 11b), it is complete. The response in Figure 11b is analogous to the one in Figure 8b in terms of total recovery of the initial stiffness: this is coherent since in the former case, the vertical displacement is almost negligible compared with the horizontal one, while in the latter case, it is absent. 

This observation translates in the fact that the multidirectional procedure for Loading Type (ii) is able to simulate the same MCR capabilities of the multidirectional procedure in case of Load Type (i), when the maximum rotation performed by the strain principal directions is large enough.

**Figure 12:**
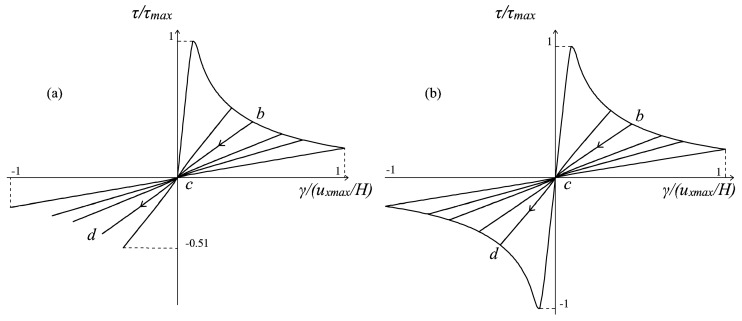
Structural response for the problem represented in Figure 5a, considering a loading history for the horizontal displacement *u_x_* composed of five cycles: (**a**) damage formulation presented in Section 2 and (**b**) multidirectional damage model.

Finally, referring to the problem of the pre-contracted panel, the case of a top displacement history *u_x_* composed of five cycles with increasing amplitude is analyzed. The ratio between the amplitude *u_x_*_1_ of the first cycle and the contraction *u_ymax_* is equal to eight, meaning that a full stiffness recovery is expected in the first cycle with the adoption of the multidirectional damage model. The responses obtained with the original damage model and with the new formulation are shown in Figure 12a,b, respectively. 

Once again, the enhanced MCR capabilities of the multidirectional procedure are evident: while in a generic unloading and reloading regime *b-c-d*, in Figure 12a, no stiffness regain is present, in Figure 12b, a clear transition from a damaged stiffness to a less damaged stiffness (the initial elastic one in the first cycle) is visible, and it is related to the switch from a damage region to another damage region, due to the rotation of the principal strain directions. From the example, it can be also deduced how the enhanced description of unilateral effects permits avoiding an underestimation of the structural performance under cyclic actions, which is instead an intrinsic problem of the original formulation. In fact, referring to Figure 12a, in the reloading stage, a peak strength unrealistically much lower than the peak strength in the loading stage is attained, and this is particularly pronounced in the first cycle. Conversely, the behavior predicted by the multidirectional damage model (see Figure 12b) is not characterized by such an asymmetry, and this is fully supported by experimental evidence on shear (masonry or reinforced concrete) panels subjected to cyclic conditions [20,21].

## 6. Conclusions

In the present paper, an enhanced version of the *d^+^*/*d^−^* model originally presented in [1], apt for cohesive-frictional materials, is proposed. The betterments regard two essential aspects:

1. the description of the orthotropy induced in the material by the degradation process is achieved by means of a consistent secant stiffness operator, whose definition is explicitly provided. To do this, the strain equivalence assumption considered in the original model is abandoned in favor of an energy-equivalent formulation, which yields both symmetry and positive definiteness in the constitutive matrix (Equation (23)). Further confirmation of the adequacy of this procedure in representing that the damage-induced orthotropy can be found in the analogy between the energy-equivalent framework and the tensor mapping procedure adopted in [35] for modelling an orthotropic behavior. From a mechanical point of view, the effects of the new *d*^+^/*d*^−^ formulation are especially visible in the lateral deformation behavior of the material, whose relevance has been generally overlooked in damage mechanics; specifically, its adoption permits simulating a reduction of Poisson’s ratio throughout the damage process, rather than considering it unrealistically constant. Moreover, having an explicit version of the secant stiffness matrix, which lacks in the original formulation, is beneficial in computational terms since it allows the implementation of the secant Picard method.
2. Microcrack closure-reopening effects are taken into account in the presence of generic cyclic conditions, especially under shear, making the model suitable for dealing with seismic actions. In fact, the impossibility of maintaining the memory of the damage directionality, due to the adoption of only the scalar variables *d^+^* and *d^−^*, is overcome with the formulation of a “multidirectional” damage model. According to this procedure, a partition of the plane (2D problems) into two regions for tensile damage and two regions for compressive damage is performed, in order to monitor separately two damage values in tension and two damage values in compression, differing for the direction in which they act. The active *d^+^* (*d^−^*) is chosen between these two tensile (compressive) damage values on the basis of the current principal directions. Moreover, the distinction between two different cyclic conditions, i.e., cyclic loadings considered alone (Type Load (i)) or preceded by not-cyclic permanent loads (Type Load (ii)) and the proposal of an ad hoc procedure for each of them add versatility to the formulation, since after loading reversal, a complete stiffness recovery, no stiffness recovery and even a partial stiffness recovery can be modelled, as demonstrated in the problem of the shear panel.

As a final remark, it has to be noticed that the new version of the *d^+^*/*d^−^* model, including the two enhancements just described, is endowed with the same algorithmic efficiency of the original one: the energy-equivalent assumption allows following, once again, a strain-based formulation (see Table 1), and the multidirectional procedure is able to maintain the memory of the degradation directionality without resorting to a tensor damage definition.

## Figures and Tables

**Table 1:** Algorithm for the *d^+^*/*d^−^* damage model based on energy equivalence.

Load increment ***n = 0***:
Set *r^+^_n_ = r^+^*_0_, *r^−^_n_ = r^−^*_0_ (definition of *r*^±^_0_ from Equation (35)), *d^+^_n_* = 0 and *d^−^_n_* = 0.
Load increment ***n***:
Compute the strain tensor ***ε****_n_*. Compute the projection operators ***Q_CW_****_n_* and ***I*** − ***Q_CW_**_n_* with the spectral decomposition of the strain tensor ***ε****_n_* (Equation (15)). Compute the elastic stress tensor ***σ_en_*** (Equation (43)). Compute *τ^+^_n_* (Equation (40)) and *τ^−^_n_* (Equation (41)). If *τ*^±^*_n_ > r*^±^*_n−_*_1_: update damage thresholds *r*^±^*_n_ = τ*^±^*_n_* and update *d*^±^*_n_* (Equations (44) and (45). If *τ*^±^*_n_ < r*±*_n−_*_1_: no updating is required, i.e., *r*^±^*_n_ = r*^±^*_n−_*_1_ and *d*^±^*_n_ = d*^±^*_n−_*_1_. Compute the operator A^*^_n_ (Equation (18)): $A_{n}^{*}=\sqrt{1-d_{n}^{+}}Q_{CWn}+\sqrt{1-d_{n}^{-}}\left(I-Q_{CWn}\right)$. Compute $\sigma_{n}$ adopting the symmetric secant operator $D_{En}$ (Equation (23)): $\sigma_{n}=A_{n}^{*}:D_{0}:A_{n}^{*}:\varepsilon_{n}$.

**Table 2:** Algorithm for the multidirectional *d^+^/d**^−^*** damage model, Type Load (i).

Load increment ***n = 0***:
Set *r*^±^_1_ = *r*^±^_2_ = *r*^±^_0_ (definition of *r*^±^_0_ from Equation (38)) and *d*^±^_1_ = *d*^±^_2_ = 0.
Load increment ***n = 1***:
Perform steps i, ii, iii and iv described in Table 1. Define the bisectors of the damage regions according to the eigenvectors of the strain tensor ***ε****_n_*: ***bisector****^+^*_1_ = ***p_max_n_**; **bisector**^+^*_2_ *= **p_min_n;_ bisector**^−^*_1_ *= **p_min_n_**; **bisector**^−^*_2_ *= **p_max_n_*** Call the subroutine “*Damage multidirectional updating* (*θ* = π/4)”. Perform steps vi and vii described in Table 1.
Load increment ***n***:
Save the strain tensor ***ε****_n_*_−1_. Perform Steps i, ii, iii and iv described in Table 1. Call the subroutine “*Damage multidirectional saving* (*θ* = π/4)”. Call the subroutine “*Damage multidirectional updating* (*θ* = π/4)”. Perform Steps vi and vii described in Table 1.

**Table 3:** Algorithm for the multidirectional *d^+^/d^−^* damage model, Type Load (ii).

Load increment ***n = 0***:
Set *r*^±^_1_ = *r*^±^_2_ = *r*^±^_0_ (definition of *r*^±^_0_ from Equation (38)) and *d*^±^_1_ = *d*^±^_2_ = 0. Set *multidir_damage* = false (logical variable which monitor the activation of the multidirectional procedure) and *r*^±^*_θn_ =* cos (*θ_min_*).
Load increment ***n = 1***:
Perform Steps i, ii, iii and iv described in Table 1. Save the strain principal directions ***p_max_n_*** and ***p_min_n_*** and define the bisectors of the damage regions: ***bisector****^+^*_1_*_=_**p_max_n_** + θ_min_*;***bisector**^+^*_2_*_=_**p_max_n_** − θ_min_* ***bisector****^−^*_1_*_=_**p_min_n_** + θ_min_*;***bisector**^−^*_2_*_=_**p_min_n_** − θ_min_* Compute the equivalent deviation quantity *τ*^±^*_θn_* according to Equation (48). If *τ*^±^*_θn_* < *r*^±^*_θ n−_*_1_ and *τ*^±^*_θn_* > cos (π/4): *r*^±^*_θ n_* = *τ*^±^*_θn_*, i.e., *θ*^±^*_r n_ = θ*^±^*_τ n_*, *multidir_damage* = true and call the subroutine “*Bisectors updating* (*θ = θ*^±^*_r n−_*_1_)”. If *τ*^±^*_θ_*_n_ > r^±^*_θ_*_n-1_ or (*τ*^±^*_θ_*_n_ < r^±^*_θ_*_n-1_ and *τ*^±^*_θ_*_n_ < cos (π/4)): r^±^*_θ_*_n_ = r^±^*_θ_*_n-1_, i.e., *θ*^±^_r n_ = *θ*^±^_r n−1_. Call the subroutine “*Damage multidirectional updating* (*θ = θ*^±^*_r n_*)”. Perform Steps vi and vii described in Table 1.
Load increment ***n***:
Save the strain tensor ***ε****_n_*_−1_. Perform Steps i, ii, iii and iv described in Table 1. Compute the equivalent deviation quantity *τ*^±^*_θn_* according to Equation (48). If *τ*^±^*_θn_* < *r*^±^*_θ n−_*_1_ : *r*^±^*_θ n_* = *τ*^±^*_θn_*, i.e., *θ*^±^*_r n_ = θ*^±^*_τ n_*, *multidir_damage* = true and call the subroutine “*Bisectors updating* (*θ = θ*^±^*_r n−_*_1_)”. If *τ*^±^*_θ_*_n_ > r^±^*_θ_*_n-1_ : r^±^*_θ_*_n_ = r^±^*_θ_*_n-1_, i.e., *θ*^±^_r n_ = *θ*^±^_r n−1_. If *multidir_damage* = false: *d^+^*_1_ = *d^+^*_2_ *= d^+^_n-_*_1_, *d^−^*_1_ = *d^−^*_2_ *= d^−^_n-_*_1_, *r^+^*_1_ = *r^+^*_2_ *=r^+^_n-_*_1_, *r^−^*_1_ = *r^−^*_2_ *=r^−^_n-_*_1_ If multidir_damage = true: call the subroutine “*Damage multidirectional saving* (*θ = θ*^±^_r n_)”. Call the subroutine “*Damage multidirectional updating* (*θ = θ*^±^*_r n_*)”. Perform Steps vi and vii described in Table 1.

**Table 4:** Constitutive properties adopted in the numerical analyses.

*E* (MPa)	*ν* (-)	*f^+^* (MPa)	*f^−^* (MPa)	*γ_e_^+^* (-)	*γ_p_^+^* (-)	*γ_e_^−^* (-)	*γ_p_^−^* (-)	*G_f_^+^* (N/mm)	*G_f_^−^* (N/mm)	*f_b_^−^*/*f^−^* (-)
1540	0.2	0.13	−3.9	1	1	0.5	1.3	0.1	10	1.15

## Appendix A

This Appendix describes the steps necessary in order to prove the consistency of the model proposed in Section 3 with regard to the second principle of thermodynamics. Specifically, in accordance with the Clausius–Duhem inequality, the positiveness of the work done by the generalized thermodynamic forces has to be demonstrated (Equation (32)). Since the rates ${\dot{d}}^{+}$ and ${\dot{d}}^{-}$of the damage variables are positive (due to the choice of the monotonically-increasing damage evolution laws; see Section 3.3), the non-negative energy dissipation is satisfied as long as the damage energy release rates are positive. The definition for these quantities, referring to the potential *ψ* expressed in (29), is:

(A1) $$-\frac{\partial \psi }{\partial d^{+}}=-\frac{1}{2}\varepsilon :\frac{\partial D_{E}}{\partial d^{+}}:\varepsilon$$

(A2) $$-\frac{\partial \psi }{\partial d^{-}}=-\frac{1}{2}\varepsilon :\frac{\partial D_{E}}{\partial d^{-}}:\varepsilon$$

Accounting for Equations (18) and (23), the derivatives of the secant stiffness operator with respect to the damage variables are:

(A3) $$\begin{array}{cc} \frac{\partial D_{E}}{\partial d^{+}}= & 2\frac{\partial A*}{\partial d^{+}}:D_{0}:A*=\\ & -\frac{1}{\sqrt{1-d^{+}}}\left[\sqrt{1-d^{+}}Q_{CW}:D_{0}:Q_{CW}+\sqrt{1-d^{-}}Q_{CW}:D_{0}:\left(I-Q_{CW}\right)\right] \end{array}$$

(A4) $$\begin{array}{cc} \frac{\partial D_{E}}{\partial d^{-}}= & 2\frac{\partial A*}{\partial d^{-}}:D_{0}:A*=\\ & -\frac{1}{\sqrt{1-d^{-}}}\left[\sqrt{1-d^{-}}\left(I-Q_{CW}\right):D_{0}:\left(I-Q_{CW}\right)+\sqrt{1-d^{+}}\left(I-Q_{CW}\right):D_{0}:Q_{CW}\right] \end{array}$$

Replacing these definitions in Equations (A1) and (A2) and recalling that ***ε^+^*** *= **Q_CW_**: **ε*** and ***ε****^−^ = (**I** − **Q_CW_**): **ε***, clearer expressions for the damage energy release rates are provided:

(A5) $$\begin{array}{cc} -\frac{\partial \psi }{\partial d^{+}}= & \frac{1}{2}\left[\varepsilon^{+}:D_{0}:\varepsilon^{+}+\frac{\sqrt{1-d^{-}}}{\sqrt{1-d^{+}}}\varepsilon^{+}:D_{0}:\varepsilon^{-}\right]=\\ & \begin{array}{c} \frac{1}{2}\left[\left(\lambda {\left(tr\varepsilon^{+}\right)}^{2}+2G\varepsilon^{+}:\varepsilon^{+}\right)+\frac{\sqrt{1-d^{-}}}{\sqrt{1-d^{+}}}\lambda tr\varepsilon^{+}tr\varepsilon^{-}\right] \end{array} \end{array}$$

(A6) $$\begin{array}{cc} -\frac{\partial \psi }{\partial d^{-}}= & \frac{1}{2}\left[\varepsilon^{-}:D_{0}:\varepsilon^{-}+\frac{\sqrt{1-d^{+}}}{\sqrt{1-d^{-}}}\varepsilon^{-}:D_{0}:\varepsilon^{+}\right]=\\ & \frac{1}{2}\left[\left(\lambda {\left(tr\varepsilon^{-}\right)}^{2}+2G\varepsilon^{-}:\varepsilon^{-}\right)+\frac{\sqrt{1-d^{+}}}{\sqrt{1-d^{-}}}\lambda tr\varepsilon^{+}tr\varepsilon^{-}\right] \end{array}$$

The quantities inside round brackets in Equations (A5) and (A6) are always positive while $\lambda tr\varepsilon^{+}tr\varepsilon^{-}$ is zero in the cases of the full tensile regime (***ε^−^***= 0) or full compressive regime (***ε^+^***= 0) and negative otherwise. Consequently, the positiveness of Equations (A5) and (A6) is assured for the situations of full tensile regime (***ε^−^***= 0) and full compressive regime (***ε^+^***= 0), while having to be proven in the other cases. In order to do this, three strain states are identified as the most critical ones for the satisfaction of the second principle of thermo-dynamics; the non-negativeness of the dissipated energy (Equation (32)) for these situations allows asserting the consistency of the present model with respect to the second principle.

- In pure shear conditions (*tr**ε^+^*** = −*tr**ε^−^**,* ***ε^+^****:**ε^+^** = (tr**ε***^+^)^2^ =***ε^−^**:**ε^−^** = (tr**ε^−^***)^2^, *d^+^ ≠* 0, *d^−^* ≠ 0), the positiveness of the quantities contained in Equations (A5) and (A6) translates in the following inequalities, respectively:
  (A7) $$\sqrt{1-d^{+}}\geq \frac{\nu }{1-\nu }\sqrt{1-d^{-}}$$
  (A8) $$\sqrt{1-d^{+}}\leq \frac{1-\nu }{\nu }\sqrt{1-d^{-}}$$
  Considering the typical mechanical parameters of a cohesive-frictional material, such as concrete or masonry, these relations among the damage variables, strictly related to the fracture energies in tension and compression (see Equations (44), (45) and (47)), are always satisfied.
- In the case of uniaxial tensile load in Direction 1 (*tr**ε^+^***= *ε*_1_ and $tr\varepsilon^{-}=-2\nu \varepsilon_{1}\sqrt{1-d^{+}}$, *d^+^ ≠* 0, *d^−^* = 0), the quantity contained in Equation (A5) is always positive and can be expressed in the following way:
  (A9) $$\frac{1}{2}\left(\lambda \varepsilon_{1}^{2}\left(1-2\nu \right)+2G\varepsilon_{1}^{2}\right)\geq 0$$
  The same results can be obtained for the case of uniaxial compressive load (*d^+^* = 0 and *d^−^* ≠ 0).
- Although the quantity $\sqrt{1-d^{+}}$ is in the denominator in Equation (A5), in the case of *d^+^* close to one, the work done by $-\frac{\partial \psi }{\partial d^{+}}$ in Equation (32) remains bounded, specifically tends to be null, because in that situation, ${\dot{d}}^{+}$ tends to zero. An analogous consideration holds for the work performed by $-\frac{\partial \psi }{\partial d^{-}}$ when *d^−^* is close to one.
